# LPS and quorum sensing protect *Pseudomonas aeruginosa* bacteria from phagocytosis and destruction by *Dictyostelium discoideum* amoebae

**DOI:** 10.1016/j.isci.2026.117912

**Published:** 2026-10-19

**Authors:** Imen Ayadi, Erwann Pain, Joseph Oddy, Otmane Lamrabet, Hitakshi Vijay, Thilo Koehler, Tania Distler, Alexandre Persat, Raphael Munoz-Ruiz, Pierre Cosson

**Affiliations:** 1 Department of Cell Physiology and Metabolism, Faculty of Medicine, University of Geneva, Geneva, Switzerland; 2 Department of Microbiology and Molecular Medicine, Faculty of Medicine, University of Geneva, Geneva, Switzerland; 3 Global Health Institute and Institute of Bioengineering, School of Life Sciences, École Polytechnique Fédérale de Lausanne (EPFL), Lausanne, Switzerland; 4 Department of Applied Biology, College of Sciences, University of Sharjah, University City, Sharjah, UAE

**Keywords:** Pseudomonas aeruginosa, virulence, LPS, Quorum sensing, Dictyostelium discoideum, amoeba, Phagocytosis, killing, ROS, O-antigen

## Abstract

Free-living amoebae like *Dictyostelium discoideum* and environmental bacteria like *Pseudomonas aeruginosa* are engaged in complex predator-prey interactions. Amoeba can notably ingest and kill bacteria. In this study, we report that three different strains of the opportunistic pathogen *Pseudomonas aeruginosa* are protected from amoebal phagocytosis. Analysis of *P. aeruginosa* mutants revealed that OSA-type lipopolysaccharides (LPS) allow these *P. aeruginosa* strains to escape phagocytosis by amoebae. Amoebae can also kill extracellularly un-ingested *P. aeruginosa* bacteria. Our results indicate that induction of the Las quorum sensing (QS) makes *P. aeruginosa* resistant to reactive oxygen species, to bacteriolytic factors secreted by *D. discoideum*, and ultimately to extracellular destruction by amoebae. Together, these observations suggest that LPS and QS probably play a key role in *P. aeruginosa* escape strategies against phagocytic amoebae in the environment and shed new light on the transition of infectious *P. aeruginosa* from the environment to the human body.

## Highlights

• *P. aeruginosa* bacteria can escape predatory *D. discoideum* environmental amoebae
• *P. aeruginosa* LPS exhibit O antigens resistant to phagocytosis by amoebae
• *P. aeruginosa* are resistant to bacteriolytic effectors secreted by amoebae
• The bacterial Las quorum-sensing controls resistance to bacteriolytic effectors

## Graphical abstract

**Figure:**
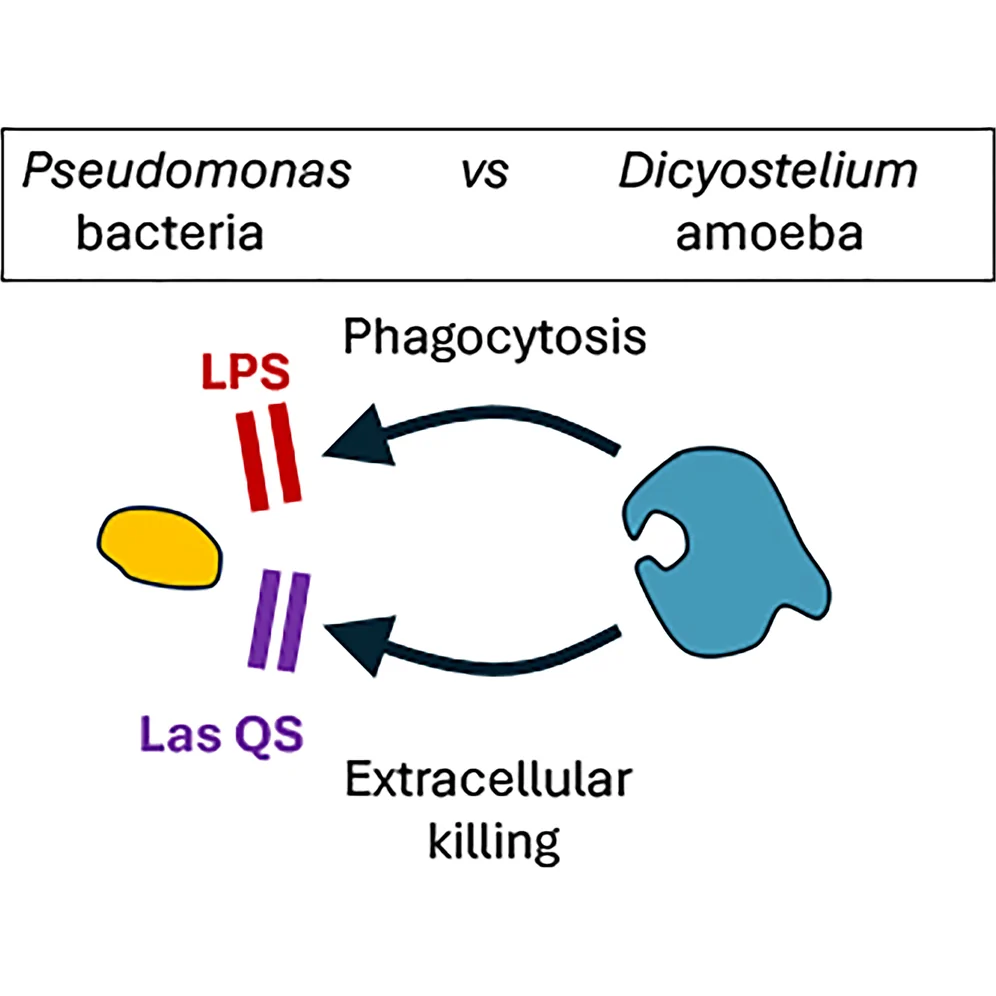

## Teaser

Cell biology; Microbial physiology; Proteomics

## Introduction

Many bacterial pathogens reside primarily in the environment. Although they exhibit virulence mechanisms allowing them to mount infections in human patients, environmental pathogens are mostly under selective pressure to survive and thrive in the environment, where they are faced with predatory phagocytic amoebae. The predator-prey relationship between bacteria and amoebae in the environment is one of the key evolutionary pressures leading to the emergence of virulence traits in bacteria.^1^^,^^2^^,^^3^

*P. aeruginosa* is an opportunistic human pathogen isolated from humid environments rich in organic matter. *P. aeruginosa* may cause acute as well as chronic infections and poses a therapeutic challenge due to the rapid emergence of antibiotic resistance mechanisms. It is one of the major causes of opportunistic infections in immunocompromised as well as in cystic fibrosis (CF)^4^ patients, where it represents a highly prevalent microbe in adult CF-patients.^5^ The pathogenic potential of *P. aeruginosa* in infected patients is achieved by a collection of virulence factors as reviewed previously,^6^^,^^7^^,^^8^ which mediate bacterial traits such as motility, adhesion, and resistance, including lipopolysaccharides (LPS), outer membrane proteins, alginate and other biofilm components, a flagellum, and type IV pili. In addition, *P. aeruginosa* uses mainly type 1 and type 2 secretion systems to export a variety of proteolytic enzymes in the extracellular medium, while the type 3 (T3SS) and type 6 (T6SS) secretion systems inject bacterial effectors directly in the cytosol of host cells. Finally, three quorum-sensing (QS) systems (Las, Rhl, and Pqs) modulate the expression of a subset of virulence genes as a function of cell density.

*D. discoideum* is a professional phagocyte naturally feeding on bacteria in the soil. It is a powerful model to study host-pathogen interactions. It has notably been used as a tractable model to study the mechanisms developed by phagocytic cells to eliminate bacteria,^9^^,^^10^ for example, to determine which phagosomal proteins are used to kill different species of bacteria.^11^ *D. discoideum* has also been used as a model host to study the virulence mechanisms developed by bacteria to survive their encounter with phagocytic cells. Previous studies showed notably that *P. aeruginosa* uses the same virulence mechanisms against mammalian phagocytes and *D. discoideum* amoebae.^12^^,^^13^

In a previous study,^14^ we reported that *D. discoideum* eliminates *P. aeruginosa* by making use of two different mechanisms. First, the amoeba can ingest *P. aeruginosa* bacteria and kill them intracellularly. Second, *D. discoideum* also secretes effectors capable of lysing uningested *P. aeruginosa* bacteria extracellularly. These two elimination strategies rely on different effectors, Kil2 being necessary for efficient intracellular destruction, while AplA and NoxA participate in extracellular destruction. AplA is a lytic effector secreted by *D. discoideum*, and NoxA is the only superoxide-producing NADPH oxidase expressed in the vegetative amoeba. It is important for *D. discoideum* to be able to kill extracellular *P. aeruginosa* because the same study showed that *P. aeruginosa* can largely escape ingestion by *D. discoideum*. Importantly, this study was performed using a non-pathogenic *P. aeruginosa* strain lacking two major QS systems (Las and Rhl).

Our aim in the current study was to analyze which molecular mechanisms allow *P. aeruginosa* to escape intracellular and extracellular destruction by *D. discoideum*. For this, we analyzed *P. aeruginosa* mutants defective for specific virulence factors. We observed the fate of these bacterial mutants when confronted with *D. discoideum*. Our results indicate that LPS and the Las QS system play key roles in protecting *P. aeruginosa* from killing by *D. discoideum* amoebae.

## Results

### OSA-type LPS protect *P. aeruginosa* from phagocytosis

We first attempted to identify the molecular mechanisms preventing efficient phagocytosis of *P. aeruginosa* by *D. discoideum* amoebae. To measure the ability of *D. discoideum* to ingest *P. aeruginosa*, we mixed GFP-expressing *P. aeruginosa* PAO1 (referred to as wild-type [WT] in this study) with *D. discoideum*. As previously described,^14^ when *D. discoideum* establishes a contact with a *P. aeruginosa* bacterium, it sometimes ingests the bacterium (Figure 1A). Ingested bacteria remain fluorescent in *D. discoideum* phagosomes for a few minutes until they are destroyed, an event which is visualized by the disappearance of the GFP fluorescence (Figure 1A). In other instances, after a few minutes of contact, *D. discoideum* releases the uningested bacteria and moves away from it (Figure 1B). We quantified how efficiently *P. aeruginosa* were ingested by measuring the percentage of bacteria ingested following a contact with a *D. discoideum* cell. WT *P. aeruginosa* PAO1 were poorly ingested (17%; Figure 1C, WT). We then analyzed bacterial mutants defective for the synthesis of the common polysaccharide antigen (CPA, formerly A-band) or the O-side chain specific antigen (OSA; formerly B-band). We also tested mutants deficient in the Las or Rhl QS systems (l*asR* knockout [KO] or *rhlR* KO, respectively). We specifically assessed the presence of OSA LPS by staining bacteria with the AE404 antibody^15^ (Figure S1A) or by purifying LPS and analyzing them by electrophoresis (Figure S1B). As expected, OSA-type O-antigens were lost only in PT1478 and PT1479 strains, and expression of OSA-type O-antigens was not visibly modified by the inactivation of Las and Rhl QS systems. Only mutants lacking the OSA LPS were ingested efficiently by *D. discoideum* (Figure 1C). *P. aeruginosa* mutants lacking CPA LPS, Las, or Rhl QS systems, were still poorly ingested (Figure 1C), indicating that these bacterial factors did not inhibit phagocytosis of *P. aeruginosa* by *D. discoideum*. A more extended collection of *P. aeruginosa* mutants (including notably mutants in the type 3 and type 6 secretion systems) was also tested, and no significant increase in the rate of phagocytosis was observed (Figure S2).

**Figure 1:**
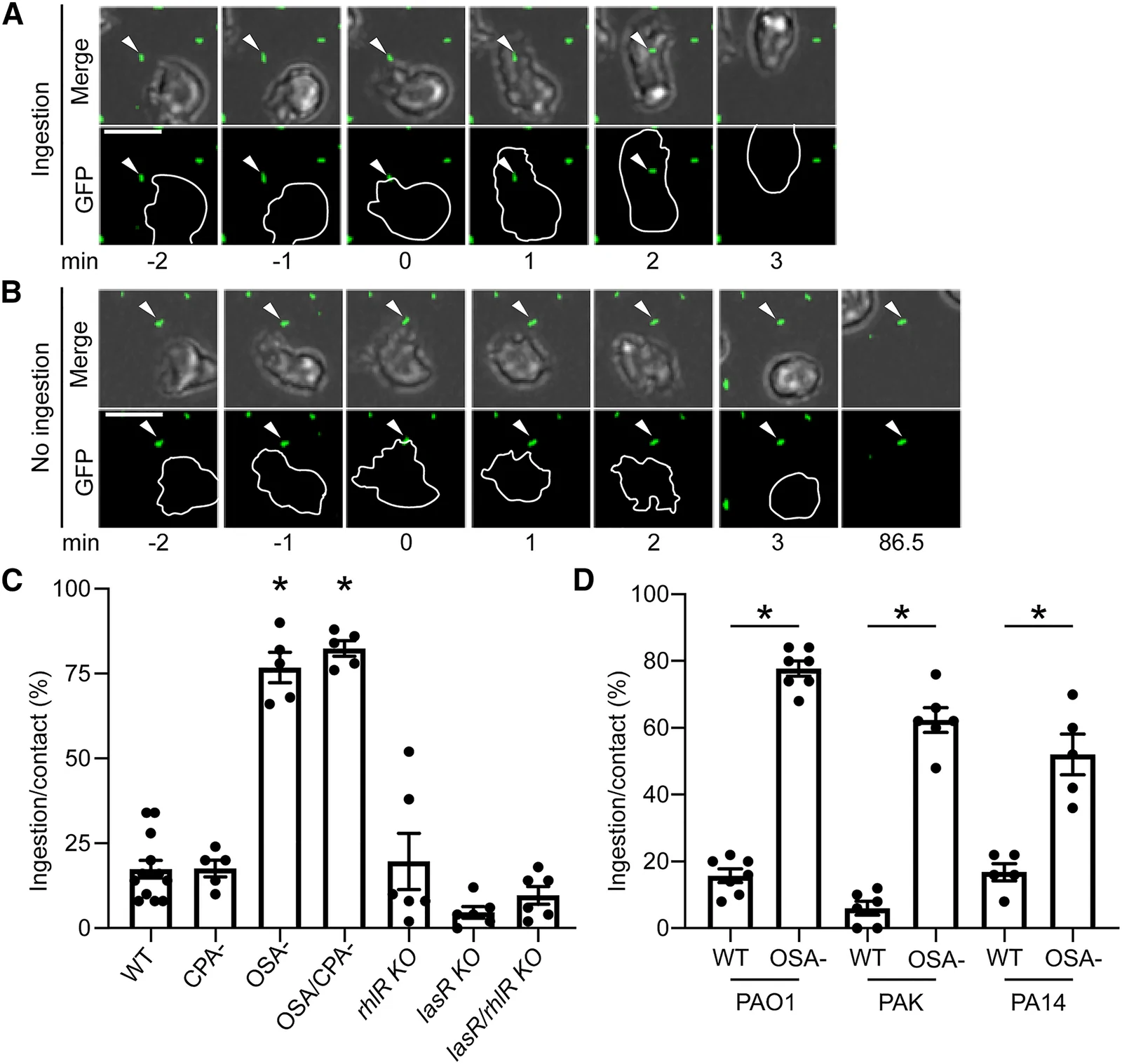
OSA LPS protect *P. aeruginosa* from phagocytosis by *D. discoideum* (A) Ingestion and intracellular destruction of a *P. aeruginosa* bacteria by a *D. discoideum* amoeba. The top row shows consecutive superimposed phase contrast and fluorescence images of a *P. aeruginosa* bacteria (white arrowheads) ingested and destroyed within a *D. discoideum* cell. In the bottom row, the phase contrast image is replaced with a white line indicating the outline of the *D. discoideum* cell. Scale bars, 10 μm. (B) A *D. discoideum* cell establishes a contact with a *P. aeruginosa* bacterial cell, without ingesting it, then moves away from the bacteria. Scale bars, 10 μm. (C) Phagocytosis of PAO1 WT and mutant bacteria was analyzed. For each bacterial cell, the percentage of contacts with *D. discoideum* resulting in ingestion was determined (mean ± SEM; *n* = 250 events analyzed, *N* = 5 independent experiments). Stars indicate mutants that are significantly more phagocytosed than WT bacteria (∗*p* < 0.05; Kruskal-Wallis test followed by Dunn’s multiple comparison). Mutant strains lacking OSA-type LPS (OSA-) were more efficiently phagocytosed than WT PAO1, mutants lacking CPA LPS (CPA-) were not. (D) Three different strains of *P. aeruginosa* (PAO1, PAK, and PA14) were analyzed. For each strain, a mutant with defective synthesis of OSA LPS was analyzed in parallel. All three *P. aeruginosa* strains were highly resistant to phagocytosis, and alteration of OSA LPS increased significantly their ingestion by amoebae (∗*p* < 0.05; Mann-Whitney test; *N* = 5–7 independent experiments).

OSA-type LPS are highly variable between different bacterial species and between different strains within the same bacterial species, thereby defining O-serotype classes. The PAO1 strain belongs to the O5 serotype characterized by (D-ManNAc3NAmA/D-ManNAc3NAcA/D-FucNAc) repeats.^16^ For comparison, we also tested the PAK strain, which belongs to the O6 serotype (L-Rha/D-GalNAc3OAcAN/D-GalNFoA/D-QuiNAc repeats) and the PA14 strain which belongs to the O19 serotype (L-Rha2OAc/L-GalNAcA/D-QuiNAc repeats). Remarkably, like *P. aeruginosa* PAO1, PAK and PA14 strains were poorly ingested by *D. discoideum* (Figure 1D). This observation indicates that resistance to phagocytosis is a common trait of *P. aeruginosa* isolates. Mutants of PAO1, PAK, and PA14 devoid of OSA-type LPS were more phagocytosed than the corresponding parental strain (Figure 1D). These results indicate that OSA-type LPS from three different serotypes protect *P. aeruginosa* from phagocytosis by *D. discoideum* cells.

We then tested if OSA-type LPS also protect *P. aeruginosa* from phagocytosis by mammalian cells. For this, we incubated differentiated THP1 macrophages with WT or OSA^−^ *P. aeruginosa* for 1 h, then washed and incubated in the presence of gentamycin to kill uningested bacteria. The macrophages were then lysed and the live intracellular bacteria grown on LB plates and counted. Macrophages ingested WT and OSA^−^ *P. aeruginosa* with the same efficacy (Figure 2), suggesting that OSA-type LPS do not protect *P. aeruginosa* from phagocytosis by THP1 macrophages.

**Figure 2:**
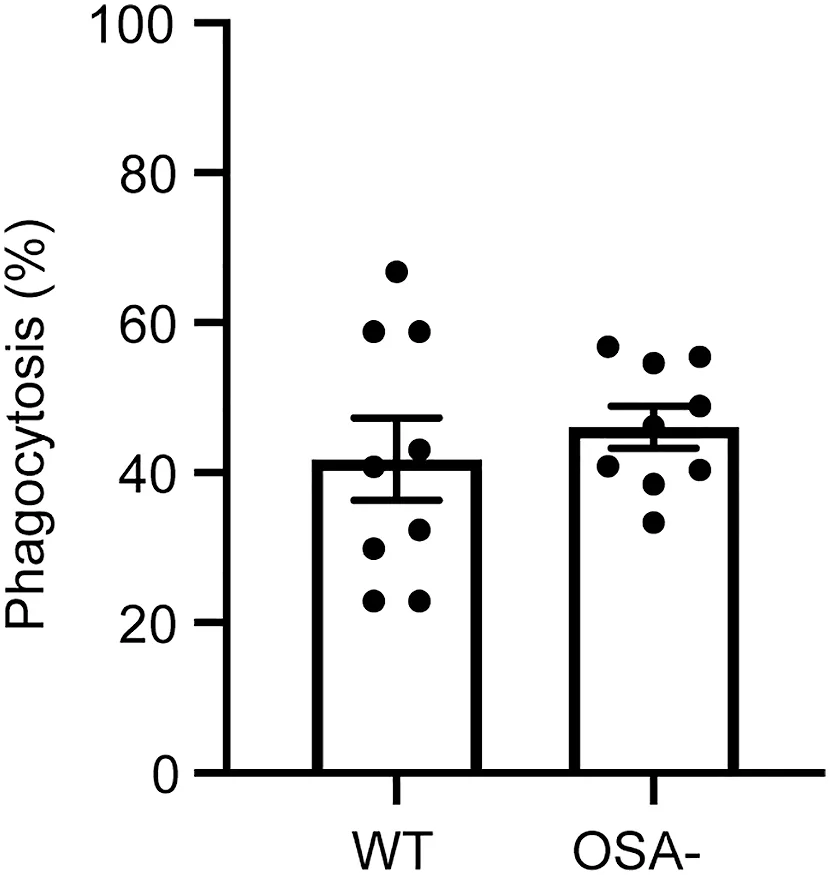
OSA LPS do not protect *P. aeruginosa* from phagocytosis by THP1 macrophages *P. aeruginosa* PAO1 bacteria (WT or OSA-) were incubated for 1 h with differentiated THP1 macrophages. The extracellular bacteria were then washed and selectively killed with gentamycin, and the intracellular bacteria plated on LB agar plates, grown overnight, and counted. The percentage of bacteria ingested by the THP1 macrophages was determined in 3 replicates in 3 independent experiments. Compared with WT bacteria, we did not observe a significant increase in the phagocytosis of mutant bacteria lacking OSA-type LPS (OSA-).

LPS could protect *P. aeruginosa* bacteria from phagocytosis by *D. discoideum* either by preventing recognition of bacteria by *D. discoideum* or by generating an anti-phagocytic signal in *D. discoideum*. To discriminate between these two possibilities, we mixed GFP-expressing *P. aeruginosa* PAO1 with RFP-expressing *Klebsiella pneumoniae* (KpGe strain) (Figure 3). Previous studies have shown that this strain of *K. pneumoniae* is efficiently ingested and killed by *D. discoideum*.^14^ Remarkably, when a *K. pneumoniae* bacteria and a *P. aeruginosa* bacteria were located next to each other, an incoming *D. discoideum* ingested the *K. pneumoniae* bacteria efficiently, and not the *P. aeruginosa* (Figure 3A). Quantification of several independent experiments indicated that the phagocytosis of *K. pneumoniae* bacteria was not hampered by the presence in their immediate vicinity of a *P. aeruginosa* bacteria (Figure 3B). This observation suggests that defective phagocytosis of *P. aeruginosa* is not caused by an inhibitory signal, which would then also inhibit phagocytosis of the nearby *K. pneumoniae* bacteria. Rather, *P. aeruginosa* are not ingested because they are not bound by *D. discoideum* phagocytic receptors, due to their protective OSA-type LPS layer.

**Figure 3:**
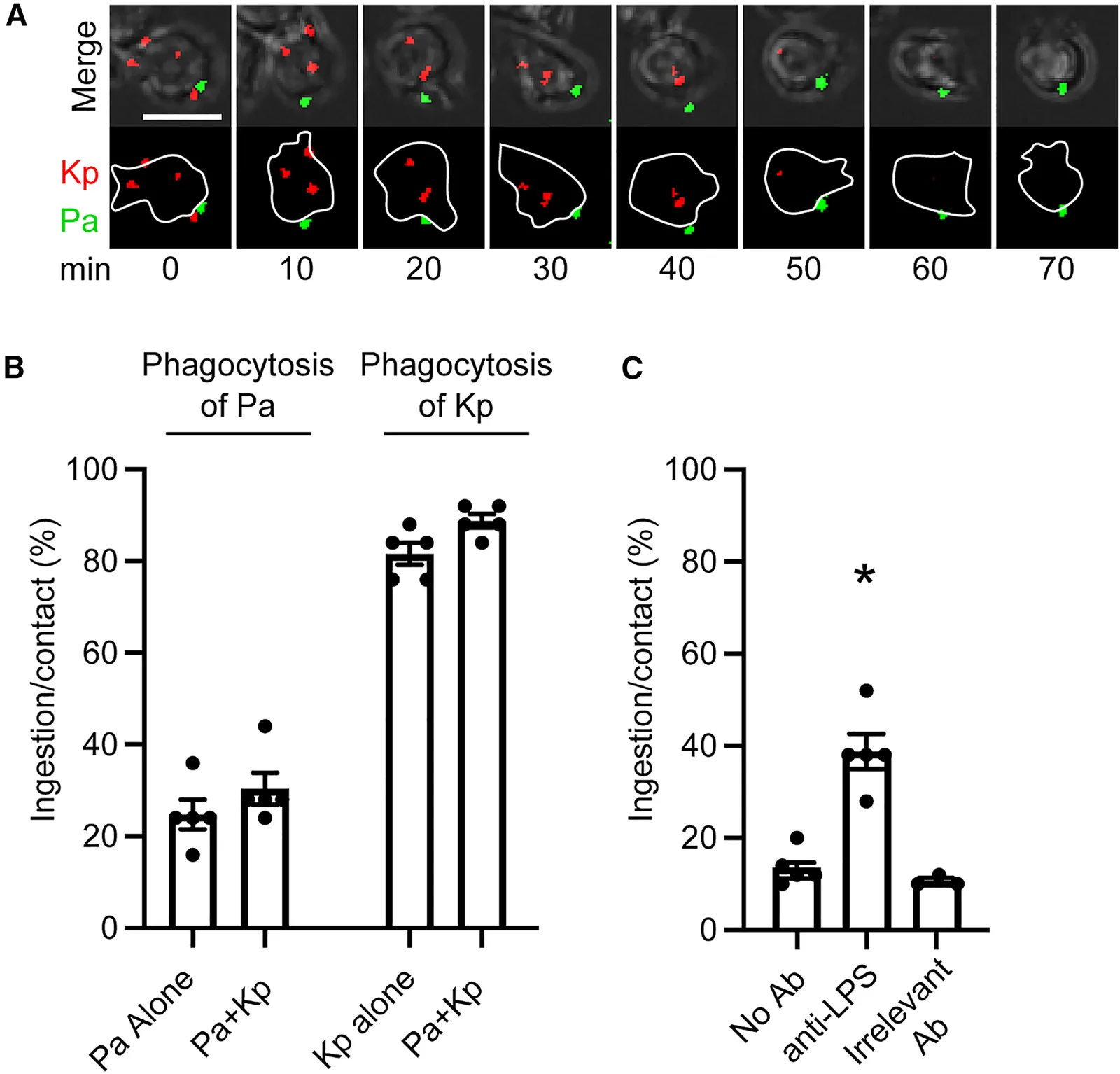
*D. discoideum* cells selectively ingest *K. pneumoniae* and not *P. aeruginosa* bacteria *D. discoideum* cells were incubated with a mixture of GFP-expressing *P. aeruginosa* (Pa, green) and mCherry-expressing *K. pneumoniae* (Kp, red). (A) In the example shown, a *D. discoideum* amoeba encountered two closely apposed bacteria and selectively ingested the *K. pneumoniae* and not the *P. aeruginosa*. While close contact was observed between the amoeba and the *P. aeruginosa* bacteria over a period of 70 min, this did not result in ingestion of the bacteria. Scale bars, 10 μm. (B) Ingestion by *D. discoideum* of *P. aeruginosa* and *K. pneumoniae* was quantified when only *P. aeruginosa* (respectively, *K. pneumoniae*) was present (Pa alone; respectively, Kp alone), or when *P. aeruginosa* was mixed with *K. pneumoniae* (Pa+Kp). (C) *P. aeruginosa* was incubated with an anti-LPS antibody (anti-LPS), an irrelevant antibody (Irrelevant Ab), or no antibody (No Ab) before its incubation with *D. discoideum*. Bacteria coated with anti-LPS antibodies were ingested more efficiently than uncoated bacteria (∗*p* < 0.05; Mann-Whitney test; *N* = 5 independent experiments).

*D. discoideum* exhibits phagocytic receptors capable of binding with very low specificity a wide range of hydrophilic surfaces (discussed in,^17^ see also discussion). If inefficient phagocytosis of *P. aeruginosa* by *D. discoideum* reflects the fact that *P. aeruginosa* LPS are not recognized by *D. discoideum* phagocytic receptors, then coating the surface of *P. aeruginosa* with antibodies should provide a proteic surface which can be recognized by *D. discoideum* receptors. To test this possibility, we used anti-LPS antibodies to decorate the surface of *P. aeruginosa* PAO1. When bacteria were pre-incubated with anti-LPS antibodies, their ingestion by *D. discoideum* amoebae was significantly increased (Figure 3C). Incubation with an irrelevant antibody, which does not attach to the surface of *P. aeruginosa*, did not increase phagocytosis of *P. aeruginosa* (Figure 3C). This observation further confirms that inefficient phagocytosis of *P. aeruginosa* is mostly caused by the fact that *D. discoideum* phagocytic receptors do not bind the OSA-type LPS of *P. aeruginosa*.

### Induction of Las QS protects *P. aeruginosa* from extracellular lysis by *D. discoideum*

As previously reported,^14^ *D. discoideum* is able to lyse uningested *P. aeruginosa* extracellularly, especially following a direct contact (Figure 4A). The efficacy with which uningested bacteria are lysed can be determined experimentally and plotted as a survival curve following contact (Figure 4B). Upon contact with *D. discoideum*, a small fraction of WT *P. aeruginosa* were lysed extracellularly (≈10%; Figure 4B). We then repeated the same experiment with several isogenic *P. aeruginosa* strains (Figures 4B and 4C). The *rhlR* KO strain was lysed extracellularly more efficiently than WT bacteria (Figures 4B and 4C). The effect was even more pronounced for *lasR* and *rhlR*/*lasR* mutants (Figures 4B and 4C). Virtually identical phenotypes were observed for lasI and rhlI/lasI mutants (Figure S3). On the contrary, *P. aeruginosa* mutants lacking OSA or CPA LPS were as resistant as WT bacteria to extracellular lysis. These results suggest that genes regulated by the Rhl and Las QS systems protect *P. aeruginosa* from extracellular destruction by *D. discoideum*. The fact that lysis of *lasR* mutant bacteria was as efficient as lysis of *rhlR*/*lasR* mutant bacteria suggests further that LasR is the main modulator of *P. aeruginosa* resistance to extracellular destruction.

**Figure 4:**
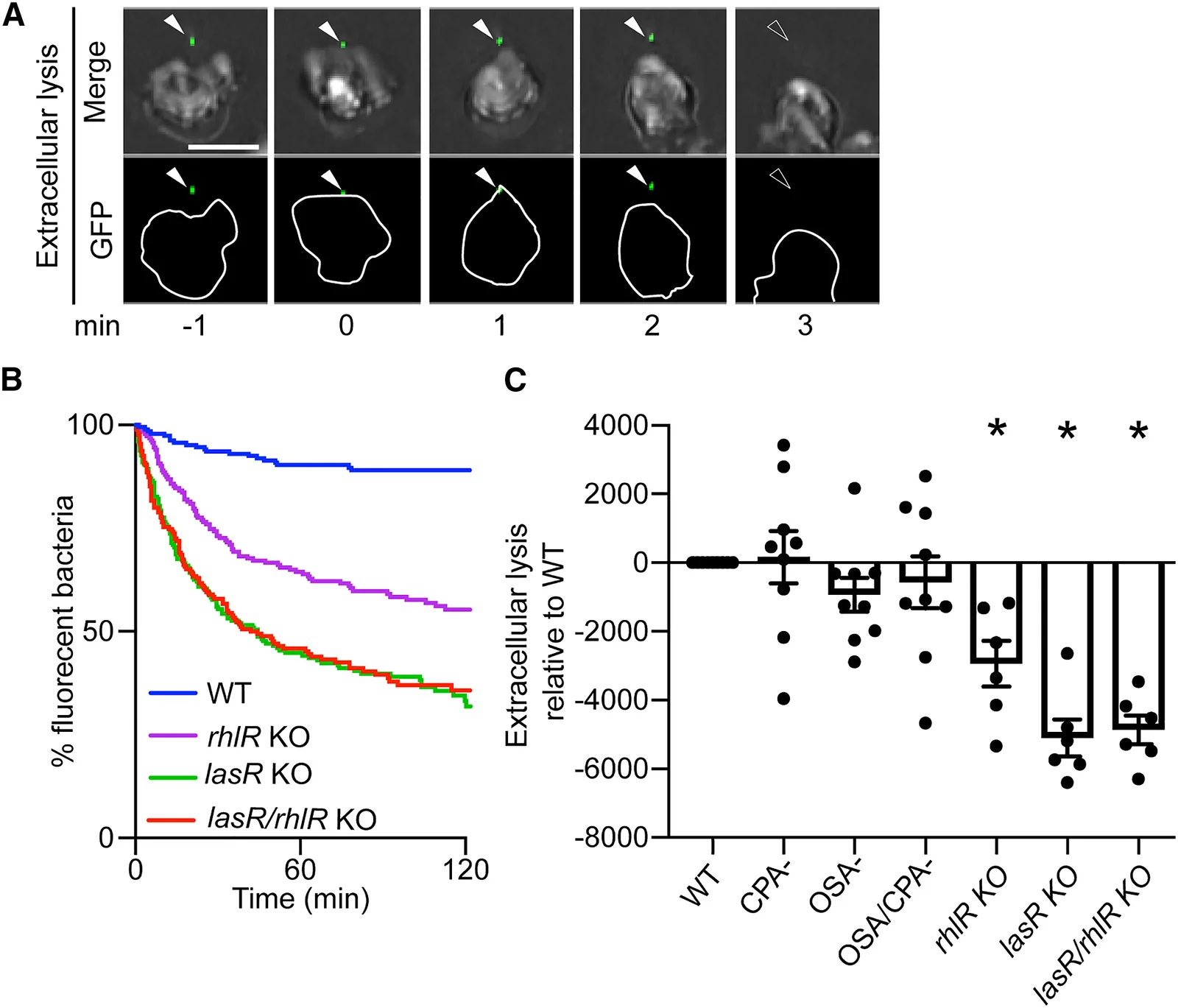
QS systems protect *P. aeruginosa* from extracellular lysis by *D. discoideum* (A) *D. discoideum* cells were mixed with fluorescent *P. aeruginosa* bacteria and observed by live microscopy, as described in Figure 1. In the example shown here, *D. discoideum* moves toward *P. aeruginosa* (white arrowheads), touches it, and leaves it. A few minutes after, the bacterium lyses (empty arrowhead). Scale bars, 10 μm. (B) Survival of extracellular *P. aeruginosa* following contact with *D. discoideum*. For each bacteria analyzed, time 0 indicates the time when it was touched by the amoeba, and GFP-extinction is concomitant with bacterial lysis. The cumulative survival curve of bacteria is shown. WT *P. aeruginosa* are poorly lysed extracellularly contrary to QS mutants. (*n* = 180 bacteria; *N* = 6 independent experiments). (C) The area under the survival curve^18^ was determined in individual experiments and normalized to WT. Mutant bacteria lacking Rhl and/or Las QS were lysed extracellularly faster than WT bacteria. Mutant bacteria lacking CPA LPS (CPA-) or OSA-type LPS (OSA-) were not. Mean ± SEM. ∗*p* < 0.05; Kruskal-Wallis test followed by Dunn’s multiple comparison test.

We then selected a number of genes that were previously shown to be induced by the las QS system^19^ and tested if genetic inactivation of these genes increased sensitivity of *P. aeruginosa* to extracellular destruction. We also tested in a similar manner several known virulence genes such as genes involved in production of the type 3 (T3SS) or type 6 (T6SS) secretion systems or genes controlling biofilm formation, exotoxin or siderophore production or bacterial motility. Among all these mutants, none was significantly more lysed extracellularly than WT bacteria (Figure S4). Resistance to extracellular destruction may be either linked to another, non-tested gene, or may be the result of the synergistic action of several distinct resistance mechanisms.

### Las QS induction renders *P. aeruginosa* resistant to several bactericidal mechanisms

To further delineate how induction of Las QS renders bacteria resistant to extracellular destruction, we examined how the phenotype of *P. aeruginosa* is modified by induction of QS. We showed previously that *D. discoideum* NoxA is necessary for efficient extracellular lysis of *P. aeruginosa*,^14^ suggesting that free radicals produced by NoxA participate in extracellular lysis of *P. aeruginosa*. Consequently, we tested whether induction of QS increases resistance of *P. aeruginosa* to hydrogen peroxide (H_2_O_2_). For this, WT or mutant bacteria were grown overnight, allowing them to induce QS, then incubated with H_2_O_2_ for 30 min at room temperature, and finally plated on LB plates and incubated at 37°C to determine their viability (Figure 5). Genetic inactivation of the Las QS but not the Rhl QS, increased strongly the sensitivity of *P. aeruginosa* to H_2_O_2_ compared to WT (Figure 5). Virtually identical phenotypes were observed for lasI and rhlI/lasI mutants (Figure S3). These observations suggest that induction of the Las QS renders *P. aeruginosa* resistant to killing by ROS.

**Figure 5:**
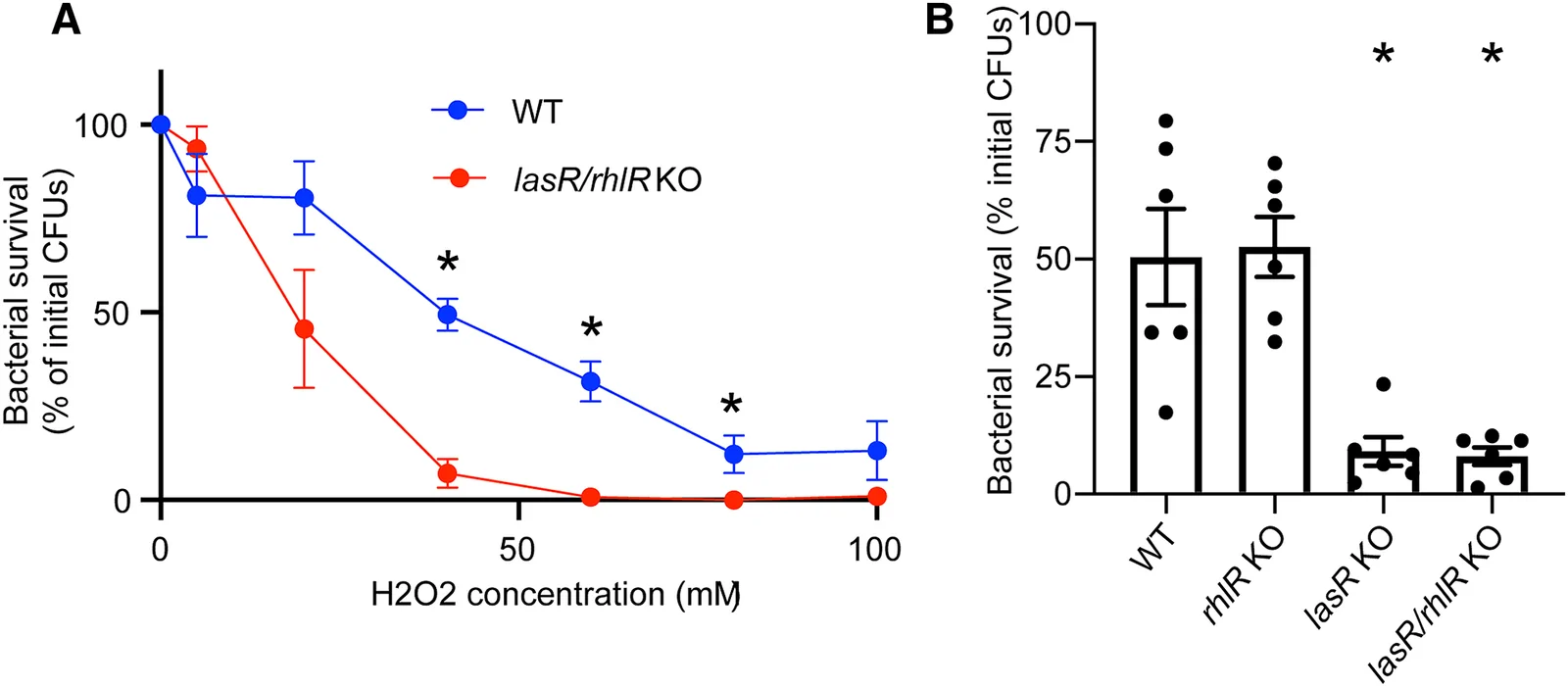
Las QS protects *P. aeruginosa* from killing by H_2_O_2_ (A) *P. aeruginosa* were incubated with variable amounts of H_2_O_2_ for 30 min then plated on LB-agar to determine their viability. (B) *P. aeruginosa* were incubated with 50 mM H_2_O_2_ for 30 min then plated on LB-agar to determine their viability. Mutants of *P. aeruginosa* lacking LasR QS were more efficiently killed by H_2_O_2_ than WT bacteria. ∗*p* < 0.05, Friedman test followed by Dunn’s multiple comparison, *N* = 6 independent experiments. CFU, colony-forming unit.

In addition to NoxA, secreted AplA was shown previously to be necessary for efficient extracellular lysis of *P. aeruginosa*.^14^ In order to test whether induction of Las QS increased resistance of *P. aeruginosa* to secreted *D. discoideum* bacteriolytic effectors, we collected the proteins secreted by *D. discoideum* cells. We then incubated *P. aeruginosa* with *D. discoideum*’s secreted proteins and assessed the integrity of bacteria by time-lapse microscopy for 2 h (Figure 6A). WT and *rhlR* KO bacteria were not efficiently lysed by exposure to secreted amoebal proteins (Figures 6B and 6C). On the contrary, *lasR* KO and *rhlR*/*lasR* KO bacteria were lysed efficiently by the secreted proteins of *D. discoideum* (Figures 6B and 6C). These observations indicate that induction of the Las QS increases the resistance of *P. aeruginosa* to lytic effectors secreted by *D. discoideum*.

**Figure 6:**
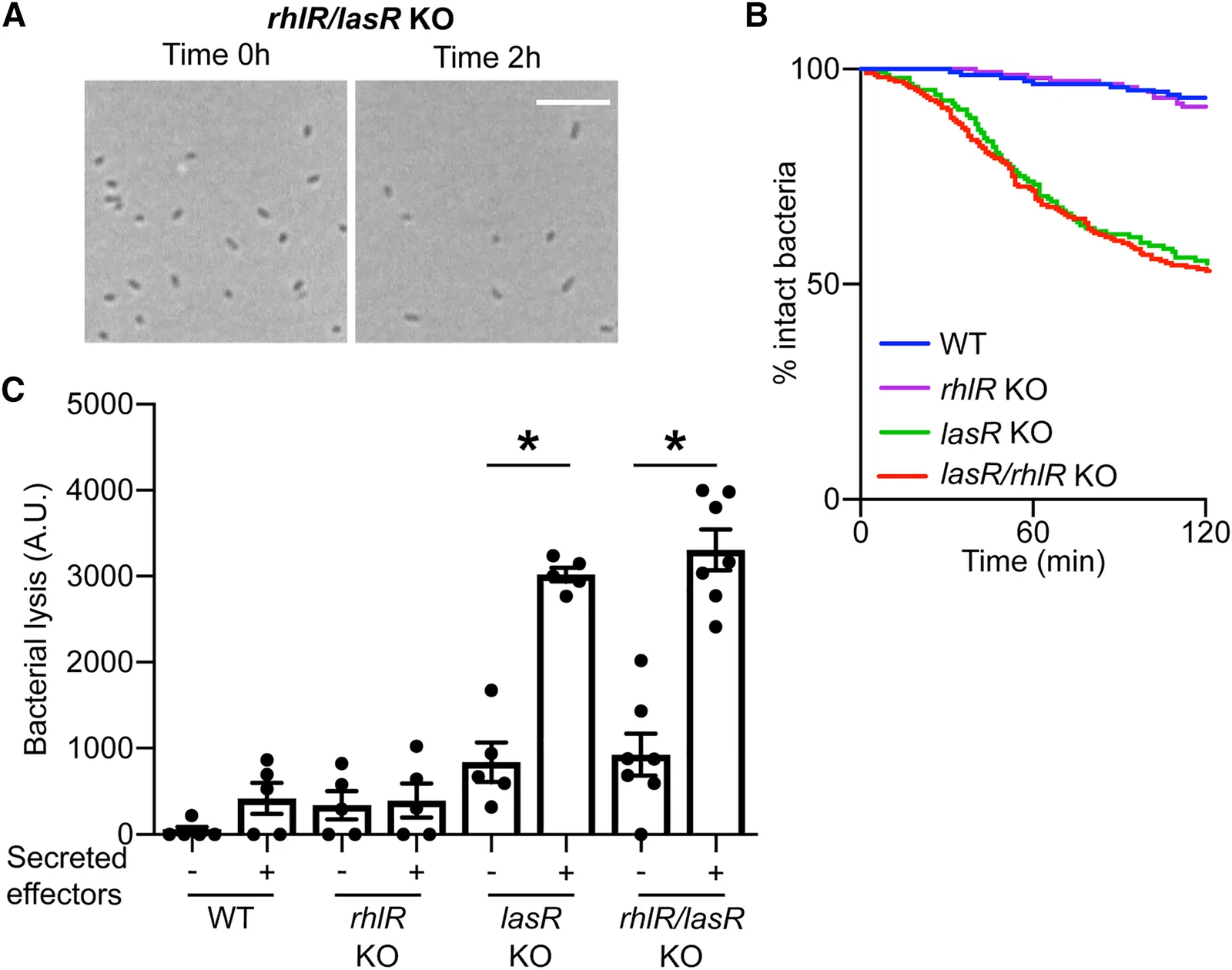
Las QS protects *P. aeruginosa* from lysis by *D. discoideum* secreted proteins *P. aeruginosa* was mixed with the amoeba secreted proteins and imaged for 2 h. (A) Phase contrast pictures showing *rhlR*/*lasR* KO *P. aeruginosa* exposed to *D. discoideum* secreted proteins for 2 h. In this example, 12/21 bacteria (57%) were lysed. Scale bars, 10 μm. (B) Survival curve of *P. aeruginosa* incubated with *D. discoideum* secreted proteins. Only *lasR* KO and *rhlR*/*lasR* KO *P. aeruginosa* were lysed efficiently. (WT: *n* = 150 events analyzed; *rhlR* KO: *n* = 150; *lasR* KO: *n* = 150; *rhlR*/*lasR* KO: *n* = 210; *N* = 5 [WT, *rhlR* KO, and *lasR* KO]; or 7 [*rhlR*/*lasR* KO] independent experiments). (C) Quantification of *P. aeruginosa* lysis in phosphate buffer (−) or in the presence of *D. discoideum* secreted proteins (+). Mean ± SEM; ∗*p* < 0.05; Mann-Whitney test, *N* = 5 or 7 independent experiments as described in (B). A.U., arbitrary units.

Resistance to free radicals and to secreted bacteriolytic factors could reflect a decreased accessibility of the bacterial membrane to extracellular factors. To test this hypothesis, we measured the accessibility of the bacterial membrane using the hydrophobic fluorescent probe 1-N-phenylnaphthylamine (NPN) as previously described.^20^ When NPN inserts in the bacterial membrane, its fluorescence strongly increases.^21^ NPN inserted poorly in the membrane of WT *P. aeruginosa* as illustrated by the resulting low level of fluorescence (Figure 7), more efficiently in the membrane of *rhlR* KO, and even more efficiently in the membrane of *lasR* KO and *rhlR*/*lasR* KO bacteria. This observation suggests that the induction of Las QS limits the accessibility of the bacterial membrane to external agents. This effect may explain at least in part how induction of QS renders *P. aeruginosa* more resistant to free radicals, bacteriolytic factors, and ultimately to extracellular lysis by *D. discoideum* cells.

**Figure 7:**
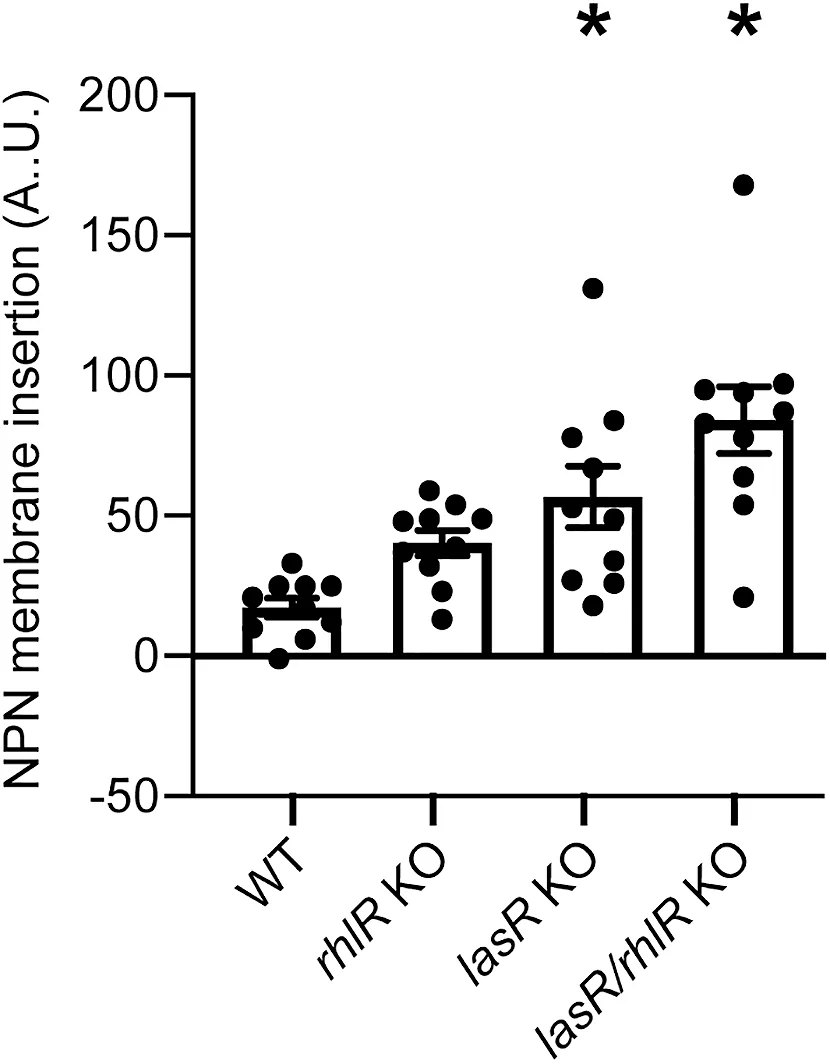
Induction of QS decreases accessibility of the bacterial membrane The accessibility of the bacterial membrane was assessed using NPN, which becomes fluorescent once incorporated in bacterial membranes. The membrane of WT bacteria was poorly accessible to NPN. In the *lasR* QS mutant, accessibility of the membrane to NPN increased significantly. A.U., arbitrary unit. Mean ± SEM; ∗*p* < 0.05, Kruskal-Wallis test followed by Dunn’s multiple comparison, *N* = 10 independent experiments. A.U. arbitrary units.

### Proteomic analysis of *D. discoideum* secreted proteins

To establish a list of putative lytic effectors secreted by *D. discoideum*, we performed a proteomic analysis of *D. discoideum* secreted proteins. A total of 205 proteins were identified (Figure 8A; Table S1). Six proteins were not encoded by the *D. discoideum* genome (e.g., human keratin) and are likely contaminants that were not considered further. Of the remaining 199 proteins, 22 did not exhibit a signal sequence and are unlikely to be authentic secreted proteins. Of the 177 *D. discoideum* proteins exhibiting a signal sequence, 47 had no known function, at least 21 were putative lytic proteins (saplip or bad proteins), and at least 63 were degradative enzymes (lipases, glycosidases, nucleases, and proteases). As expected, we identified AplA among the secreted proteins. We also compared this list of secreted proteins to the 166 *D. discoideum* proteins previously identified in a partially purified bacteriolytic extract.^22^^,^^23^ These 166 proteins are expected to represent mostly lysosomal proteins that bear sulfate or phosphate groups. The two lists of proteins largely overlapped (Figure 8B; Table S1), identifying 101 proteins which are presumably lysosomal proteins secreted by starved *D. discoideum* cells. Interestingly, this study also identifies 65 presumptive sulfated/phosphorylated lysosomal proteins that are not secreted by starved cells, and 74 non sulfated/phosphorylated proteins that are secreted by starved *D. discoideum* cells. This analysis provides a list of putative bacteriolytic proteins which can be used as a guide for future studies.

**Figure 8:**
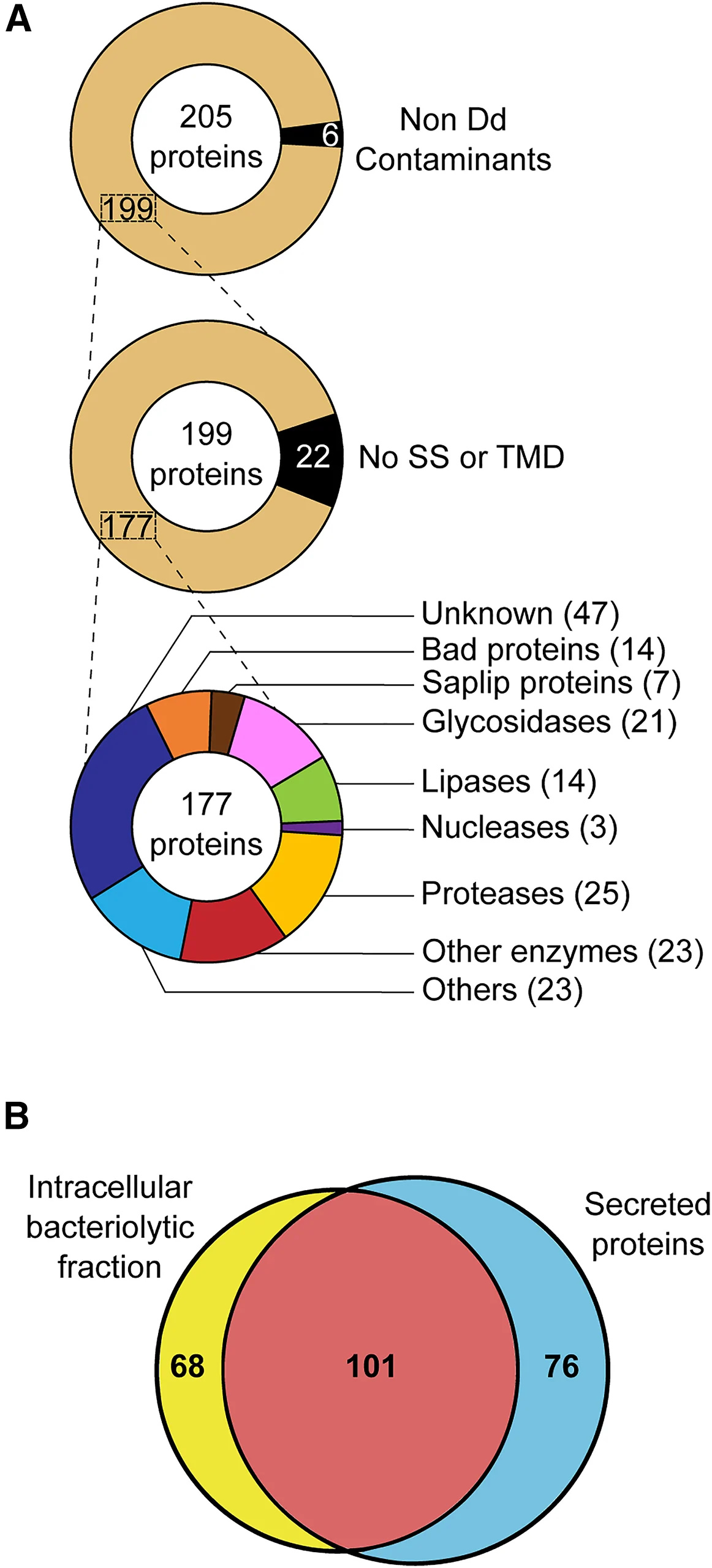
Proteomic analysis of *D. discoideum* secreted proteins Protein composition of the *D. discoideum* secretome was analyzed by mass spectrometry. (A) Among the 205 proteins identified, 6 were non-*D. discoideum* proteins (e.g., human keratin) and 22 did not display a signal sequence (SS) or a transmembrane domain (TMD). The 177 remaining *D. discoideum* proteins were classified according to their presumptive functions (Table S1). (B) Comparison of secreted proteins with proteins previously identified in partially purified fractions exhibiting bacteriolytic activities.^22^ Of the 177 secreted *D. discoideum* proteins, 101 were also found in bacteriolytic fractions. A detailed list of identified proteins is shown in Table S1.

## Discussion

In this study, we analyzed the molecular mechanisms that allow *P. aeruginosa* bacteria to survive their encounter with predatory *D. discoideum* amoebae. Our results highlight the role of bacterial LPS and QS induction in the defense of *P. aeruginosa* against predation by amoebae: the LPS of three *P. aeruginosa* strains analyzed protect them from phagocytosis by *D. discoideum*, while induction of the Las QS renders *P. aeruginosa* resistant to extracellular killing by ROS and lysosomal components released by amoebae in their vicinity. Induction of the Las QS controls the expression of many *P. aeruginosa* genes,^19^ leading us to test the role of individual genes induced by the Las QS. However, none of the Las-induced genes tested was essential for the induction of bacterial resistance to extracellular destruction by *D. discoideum*. This could reflect the fact that we did not test the critical resistance gene, or that multiple Las-induced genes cf. resistance to extracellular destruction. Previous studies have shown that the expression of catalase and superoxide dismutase genes is controlled by the QS in *P. aeruginosa*^4^ and it will be interesting in the future to explore the role of these genes in conferring resistance to extracellular killing by amoebae. Of note, resistance to extracellular destruction was likely not due to modifications of the LPS under the control of the Las QS, as evidenced by the fact that LPS appeared unchanged in *las* KO bacteria. Further studies will be necessary to determine by which mechanism induction of Las QS confers resistance to extracellular destruction. Conversely, although our previous experiments identified AplA (a putative bacteriolytic protein) and NoxA (the superoxide/H_2_O_2_ production system) as two *D. discoideum* genes implicated in the extracellular destruction of *P. aeruginosa*,^14^ it is likely that other secreted amoebal factors participate in this process. In this regard, the extensive list of proteins secreted by *D. discoideum* in the conditions of our experiments may serve in the future as a guide to identify other *D. discoideum* proteins involved in extracellular killing of *P. aeruginosa*, and possibly other bacteria.

Our results may shed new light on the biology of *P. aeruginosa* in the environment and in infected patients*. D. discoideum* amoebae can ingest bacteria very efficiently at a rate of several bacteria per minute.^24^ Several phagocytic receptors allow *D. discoideum* to ingest a wide range of particles and bacteria.^25^^,^^26^^,^^27^^,^^28^ Our results indicate that, contrary to most bacteria studied previously (e.g., *E. coli*, *K. pneumoniae*, and *B. subtilis*),^14^ three different strains of *P. aeruginosa* are very poorly phagocytosed by *D. discoideum* amoebae. For all three strains, genetic analysis suggests that OSA-type LPS prevent efficient binding of *D. discoideum* phagocytic receptors, although the three strains analyzed exhibit three different types of OSA-type LPS. This observation suggests that in its natural environment, *P. aeruginosa* may be under strong selective pressure to use OSA-type LPS that are not recognized by *D. discoideum* phagocytic receptors. An extensive analysis of *P. aeruginosa* strains exhibiting different OSA-type LPS will be necessary to establish a more detailed list of LPS conveying resistance to phagocytosis by *D. discoideum* amoebae. It will also be interesting to investigate if other bacterial species can produce LPS (or other protective layers) limiting their phagocytosis by amoebae.

During infection of a patient the role of bacterial LPS is multifaceted (reviewed in Huszczynski^29^]): the lipid A portion is recognized by host receptors such as human TLR4, activating immune cells.^30^ The outer core portion binds the CFTR protein, increasing *P. aeruginosa* ingestion by epithelial cells. Fixation of the complement system to LPS induces bacterial lysis and phagocytosis of opsonized bacteria by PMNs. The effect of LPS on phagocytosis is not clearly established: many different human cell types have been reported to ingest *P. aeruginosa* (neutrophils, dendritic cells, and epithelial cells), and *P. aeruginosa* bacteria can be recognized by phagocytic cells directly, or through the binding of opsonins (e.g., complement, surfactant proteins A and D, or antibodies binding the OSA-type LPS). In one study, loss of *P. aeruginosa* OSA LPS was seen to increase complement binding and complement-dependent phagocytosis.^31^ In another study loss of OSA LPS did not modify substantially phagocytosis of *P. aeruginosa*.^32^ Similarly, in our experiments, loss of OSA LPS did not modify substantially phagocytosis of *P. aeruginosa* by THP1 macrophages. In summary, to the best of our current knowledge, OSA-type LPS do not appear to play a significant role in protecting *P. aeruginosa* from phagocytosis by human immune cells.

Remarkably, in infected patients, *P. aeruginosa* strains often evolve from a “smooth” phenotype (OSA LPS with extended O-antigens) to a “rough” phenotype lacking OSA side chains,^6^^,^^33^ suggesting that loss of OSA LPS can be advantageous to the survival of *P. aeruginosa* in the human body, presumably because it reduces recognition by the immune system. As a tentative interpretation of these observations, *P. aeruginosa* strains growing in the environment apparently make use of LPS carrying a specific set of OSA LPS which are not recognized by the phagocytic receptors of amoebae. In infected patients these LPS cease to provide a selective advantage, and mutant strains devoid of OSA LPS can appear and expand.

The second key bacterial feature identified in this study is the induction of the bacterial QS. The QS is a well-documented mechanism to modify gene expression of *P. aeruginosa* bacteria when their cellular density increases. QS regulates expression of about 5% of the bacterial genome, notably the expression of numerous virulence genes. Our results show that induction of the two major QS systems (Las and Rhl) increases resistance to secreted *D. discoideum* antibacterial proteins, as well as resistance to ROS. Since *D. discoideum* uses superoxide and secreted lytic proteins to lyse uningested *P. aeruginosa* bacteria,^14^ these observations presumably explain the fact that QS induction also strongly increases the probability of *P. aeruginosa* to survive after a contact with an amoeba. These results explain why infecting *P. aeruginosa*, coming from the environment, very frequently exhibit functional QS systems. Remarkably, in infected patients QS-negative variants often appear and can replace the original infecting strain, suggesting that QS induction is no longer advantageous.^6^^,^^33^^,^^34^^,^^35^ These observations suggest that the ability of *P. aeruginosa* bacteria to induce QS is under positive selection pressure in the environment, where it allows bacteria to resist extracellular destruction by *D. discoideum*. In infected patients this selection pressure disappears, allowing the appearance of QS-negative variants.

In summary, LPS and QS are two major virulence factors which appear to play a critical role to ensure survival of *P. aeruginosa* in the environment where it is confronted with predatory amoebae. In the initial phase of acute infections LPS and QS increase the ability of bacteria to damage host tissues but once established and protected in biofilms LPS and QS are no longer advantageous to ensure survival of *P. aeruginosa*. This is a nice illustration of the concept of coincidental evolution where pathogen virulence is a byproduct of adaptation to other environments^1^^,^^2^: environmental adaptation of *P. aeruginosa* selects resistance traits which coincidentally act as pathogenic traits during infection of mammalian hosts.

### Limitations of the study

This work was focused on three laboratory *P. aeruginosa* strains and one strain of *D. discoideum* amoeba, and observations were made in laboratory settings. Our discussion proposes a largely speculative extrapolation to the much more complex situation in infected patients and in the environment. The study of a larger set of *P. aeruginosa* strains of diverse origins will be necessary to determine if our speculations concerning evolution of *P. aeruginosa* are relevant. In particular, our speculations on the role of LPS and QS are based on the idea that the strains studied are representative of other strains in clinical settings, a hypothesis which will need to be experimentally tested. It also remains to be seen to what extent the strains studied in this study are representative of strains found in the environment.

From another perspective, we are also speculating on the selective pressures applied by environmental amoebae onto their bacterial preys based on the premise that *D. discoideum* is representative of other environmental predators. This is certainly an oversimplification which calls for the study of how other environmental unicellular predators interact with their bacterial preys. Finally, many other selective pressures (e.g., infection with phages) are expected to apply to *P. aeruginosa*. While our observations shed new light on the role of LPS and QS in the biology of *P. aeruginosa*, many more studies will be necessary to determine to what extent they can be extrapolated to the situations found in the environment and in infected patients.

## Resource availability

### Lead contact

Requests for further information and resources should be directed to and will be fulfilled by the lead contact, Pierre Cosson (pierre.cosson@unige.ch).

### Materials availability

All unique/stable reagents generated in this study are available from the lead contact without restriction.

### Data and code availability

• All data have been deposited in Yareta and are publicly available as of the date of publication (https://doi.org/10.26037/yareta:355x2vg3ybanjey76u5bumpjay). Proteomics data are available via ProteomeXchange with identifier PXD083503.
• This paper does not report original code.
• Any additional information required to reanalyze the data reported in this paper is available from the lead contact upon request.

## Acknowledgments

This research was supported by the 10.13039/501100001711Swiss National Science Foundation, grant nos. 10.004.845 (to P.C.) and 310030_189084 (to A.P.). The funding bodies played no role in the design of the study, the collection, analysis, and interpretation of data and in writing the manuscript. We thank Alexandre Luscher for assistance with bacteriology experiments, the Proteomics facility of the Geneva Faculty of Medicine for performing proteomics analysis. We thank colleagues who provided *P. aeruginosa* strains included in this analysis, as listed in the key resources table.

## Author contributions

Conceptualization and writing – review and editing, all authors; investigation and formal analysis, I.A., E.P., J.O., O.L., H.V., T.D., A.P., R.M.-R., and P.C.; visualization, I.A. and H.V.; resources, T.K.; supervision, P.C. and A.P.; writing – original draft, I.A. and P.C.; project administration, P.C.

## Declaration of interests

P.C. and R.M.-R. are founders and shareholders of ABCD Antibodies SA from which two antibodies used in this study were bought. This company was not involved in the funding, design, conduct, or publication of this study.

## STAR★Methods

### Key resources table

**Table:** 

REAGENT or RESOURCE	SOURCE	IDENTIFIER
**Antibodies**		

Mouse AE404 anti-O5 LPS	ABCD Antibodies	ABCD_AE404; RRID: 3756908
Mouse AF291 anti-HA	ABCD Antibodies	ABCD_AF291; RRID: 3754698

**Bacterial and virus strains**		

*P. aeruginosa* PAO1 WT	Thilo Kohler	PT5
*P. aeruginosa* PAO1 WT	Joseph Lam	PT1477
*P. aeruginosa* PAO1 (CPA-/OSA-) *wbpL*	Joseph Lam	PT1478
*P. aeruginosa* PAO1 (CPA+/OSA-) *wbpM*	Joseph Lam	PT1479
*P. aeruginosa* PAO1 (CPA-/OSA+) *rmd*	Joseph Lam	PT1482
*P. aeruginosa* PAO1 *rhlR*	Thilo Kohler	PT462
*P. aeruginosa* PAO1 *lasR*	Thilo Kohler	PT498
*P. aeruginosa* PAO1 *rhlR/lasR*	Thilo Kohler	PT531
*P. aeruginosa* PAO1 *fliC (flagella)*	Thilo Kohler	PT690
*P. aeruginosa* PAO1 *D3tox (exoS, T and Y)*	Arne Rietsch	PT1720
*P. aeruginosa* PAO1 *popBD (T3SS)*	Ina Attree	PT1722
*P. aeruginosa* PAO1 *PA0087 (T6SS)*	Marek Basler	PT2267
*P. aeruginosa* PAO1 *pvd/pch (siderophore)*	Dieter Haas	PT13
*P. aeruginosa* PAO1 *pslA (biofilm)*	Anthony DiGiandomenico	MED3
*P. aeruginosa* PAO1 *lasB (LasQS-regulated)*	Manoil Lab	PA3724
*P. aeruginosa* PAO1 *pepB (LasQS-regulated)*	Manoil Lab	PA2939
*P. aeruginosa* PAO1 *aprX (LasQS-regulated)*	Manoil Lab	PA1245
*P. aeruginosa* PAO1 *aprD (LasQS-regulated)*	Manoil Lab	PA1246
*P. aeruginosa* PAO1 *aprE (LasQS-regulated)*	Manoil Lab	PA1247
*P. aeruginosa* PAO1 *aprF (LasQS-regulated)*	Manoil Lab	PA1248
*P. aeruginosa* PAO1 *pslB (LasQS-regulated)*	Manoil Lab	PA2232
*P. aeruginosa* PAK WT	Joseph Lam	PAK
*P. aeruginosa* PAK *wbpL* B-	Joseph Lam	PAK B-
*P. aeruginosa* PA14 WT	A. Luscher	PA14
*P. aeruginosa* PA14 orfN B-	A. Luscher	BAL269

**Deposited data**		

Proteomics data	ProteomeXchange	PXD083503
Experimental data	Yareta	https://doi.org/10.26037/yareta:355x2vg3ybanjey76u5bumpjay

**Experimental models: Organisms/strains**		

*D. discoideum* WT strain	Cosson lab	DH1-10

### Experimental model and study participant details

#### Cell culture and strains

*D. discoideum* DH1-10^17^ cells (referred to here as WT) were grown at 21°C in HL5 medium^36^ containing 15.6 μg/mL of tetracycline in Petri dishes and diluted every three days to remain below a density of 10^6^ cells/mL. All experiments with live *D. discoideum* cells were carried out at 21°C.

Strains of *P. aeruginosa* were described previously^18^^,^^37^^,^^38^ or sent by the Manoil Lab mutant library (UWGE, https://www.gs.washington.edu/labs/manoil/libraryindex.htm), as indicated in the key resources table. Unless otherwise specified, all the mutants were derived from the parental (WT) PAO1 strain of *P. aeruginosa*. We used two WT strains of PAO1, one from the Kohler laboratory (PT5) for all mutants generated in this group, the other from the Lam laboratory (PT1477). Both WT strains gave very similar results, referred to collectively as WT PAO1. GFP-expressing strains of *P. aeruginosa* were obtained using the plasmid pIApX2, a kind gift of Ina Attrée^39^ which conferred carbenicillin resistance. Before each experiment, GFP-expressing *P. aeruginosa* strains were grown in 3 mL SM medium containing 100 μg/mL of carbenicillin for 16 hours at 37°C with shaking.^36^ *K. pneumoniae* expressing mCherry have been described previously.^40^

### Method details

#### Phagocytosis and destruction of bacteria

The phagocytosis, intracellular or extracellular destruction of fluorescent *P. aeruginosa* bacteria in the presence of *D. discoideum* cells was visualized and measured as described previously.^14^ Briefly, 300μL of GFP-expressing bacteria from an overnight culture and 7 × 10^5^ *D. discoideum* cells were washed once in phosphate buffer (PB: 2 mM Na_2_HPO_4_ + 14.7 mM KH_2_PO_4_ pH 6.3) supplemented with 100 mM sorbitol (PB-Sorbitol). Bacteria were diluted 1:100 in PB-Sorbitol and *D. discoideum* cells were resuspended in 250 μL PB-Sorbitol. 100 μL of *D. discoideum* cells and 150 μL of diluted bacteria were deposited in a glass-bottom well (μ-slide 8-well; IBIDI). Slides were centrifuged (278 *g*, 10 min) to ensure efficient sedimentation of bacteria and cells. Movies were recorded for 2 h with a Nikon eclipse Ti2 widefield time-lapse microscope equipped with a DS-Qi2 camera. To image the whole cell volume, every 30 sec an image of a whole field (bright field and GFP fluorescence) was taken in five successive focal planes with a step size of 3 μm. These movies were blinded and analysed to determine the efficiency of phagocytosis, of intracellular and of extracellular destruction. As described previously,^14^ to measure efficiency of phagocytosis, we counted bacteria which had been touched by wandering amoebae and determined in each experiment what percentage of these bacteria were ingested. To measure kinetics of intracellular killing, for each ingested bacteria, time 0 was the time at which it was ingested, and bacterial destruction was defined as the sudden disappearance of the GFP fluorescence. We used a Kaplan-Meier estimator to calculate the rate of destruction of ingested bacteria as previously described.^11^ Extracellular destruction was determined in a similar manner by assessing the fate of uningested bacteria following a contact with a *D. discoideum* cell.^14^

To measure how coating bacteria with antibodies modify their phagocytosis by *D. discoideum*, we used the ABCD_AE404 antibody which binds the O5-antigens of PAO1 LPS^15^ to decorate the surface of PAO1 bacteria. As an irrelevant antibody we used the ABCD_AF291 antibody directed against the HA epitope tag.^41^ Both antibodies were produced as mini-antibodies with the antigen-binding portion fused to a mouse IgG2A Fc (ABCD Antibodies, Geneva, CH; https://abcd-antibodies.com). GFP-expressing bacteria were grown overnight, then 1 mL of bacteria was collected by centrifugation (6000 *g*, 3 min), washed once and resuspended in 1 mL of PB-Sorbitol containing the indicated antibody (1 μg/mL). Bacteria were collected by centrifugation, resuspended in PB-Sorbitol and mixed with *D. discoideum* cells as described above.

#### Phagocytosis of *P. aeruginosa* by macrophages

THP1 monocytes were passaged every 3 to 4 days in RPMI supplemented with 10% foetal bovine serum (FBS) and GlutaMAX. 3 days prior to infection experiments, 200,000 cells were seeded per well (24-well plate) and 100nM PMA was added for differentiation to macrophages.

Bacteria were grown to exponential phase, washed in phosphate buffered saline (PBS) and resuspended at a concentration corresponding to an optical density at 600nm (OD_600_) of 1. In each well, 5 μL of the bacterial suspension were added in 500 μL RPMI supplemented with 10% heat-inactivated FBS (ca. MOI 10). Macrophages were incubated with the bacteria for 1 h followed by a washing step and incubation with 60 μg/mL Gentamycin for an additional hour to kill extracellular bacteria. Macrophages were then washed thrice, lysed for 10 min with 0.1% Triton in PBS, and the lysate containing live bacteria plated on LB plates. Colony forming units (CFUs) were determined for the initial inoculum and cellular lysates. Experiments were performed 3 times independently (biological replicates) and each time 3 wells were infected per mutant (technical replicates). The following formula was used to determine phagocytosis efficiency:

$$\text{Phagocytosis}\;\left(\%\right)=\frac{\left(\text{CFU}\;\left(\text{lysate}\right)\right)}{\left(\text{CFU}\;\left(\text{inoculum}\right)\right)}\times 100$$

#### *D. discoideum* secreted proteins

*D. discoideum* cells were grown in a shaken suspension (100 mL in a 500 mL flask, 160 rpm) until they reached a concentration of 5 × 10^6^ cells/mL. Cells were delicately pelleted by centrifugation (237 *g*, 5 min) and washed with 1 volume of PB-Sorbitol containing 100 μM CaCl_2_. Cells were then resuspended at a final concentration of 50 × 10^6^ cells/mL in PB-Sorbitol + 100 μM CaCl_2_ and secretion was allowed for 6 h under slow shaking (100 rpm). Cells were then gently centrifuged for 3 min, at 94 *g* to recover the cell supernatant without breaking the cells. The supernatant was then further cleared by successive 3-min centrifugations at 376 *g* and 1500 g. The final supernatant was filtered (0.22 μm filter), kept at 4°C and used within one week.

Secreted proteins were identified as previously described by ESI-LC-MSMS.^23^ The mass spectrometry proteomics data have been deposited to the ProteomeXchange Consortium via the PRIDE partner repository with the dataset identifier PXD083503 and https://doi.org/10.6019/PXD083503. The full list of proteins identified is shown in Table S1, as well as its comparison with the list of intracellular proteins previously identified in partially purified fractions exhibiting bacteriolytic activities.^22^^,^^23^ For each protein, a relative abundance score (emPAI: Exponentially Modified Protein Abundance Index) is shown.

#### Lysis of bacteria by *D. discoideum* proteins

To measure lysis of bacteria by *D. discoideum* secreted proteins, 250 μL of bacteria from an overnight culture were centrifuged (6000 *g*, 3 min) and washed with 1 mL of PB-Sorbitol. Bacteria were then resuspended in 1 mL of PB-Sorbitol, diluted 1:100 in the same buffer and 50 μL of diluted bacterial suspension was deposited in each well of a 96-well plate (Greiner, μclear black cellstar plates, 655090). The plate was centrifuged (1479 × *g*, 10 min) and 150 μL of each sample (buffer or cell supernatant) was added just before initiating recording. Imaging was performed at 25°C using the microscope ImageXpress (Molecular Devices) and 1 picture was taken every min for 2 h.

For analysis, 30 bacteria were randomly chosen. Bacteria moving out of the frame were not taken into consideration. The time when bacteria were lysed was noted. Bacterial survival was calculated using a Kaplan-Meier estimator and the area over the survival curve was used for statistical analysis.

#### Accessibility of the *P. aeruginosa* membrane

We measured the accessibility of the bacterial membrane using the hydrophobic fluorescent probe 1-N-phenylnaphthylamine (NPN; Sigma-Aldrich) as previously described.^20^ When NPN inserts in the bacterial membrane, its fluorescence strongly increases.^21^

A concentrated solution of NPN (0.5 mM) was prepared freshly the day of the experiment in acetone in a glass tube and protected from light. The concentrated solution was diluted in HEPES buffer (5 mM, pH 7.2) to a concentration of 40 μM. Bacteria from an overnight bacterial culture were centrifuged (6000 *g*, 3 min), washed in 1 mL HEPES buffer, resuspended in 1.5 mL HEPES buffer (OD_600_ = 0.15), and used as described below in a 96-well plate (cell culture microplate, PS, F-Bottom, black, Greiner bio-one). Condition A: 200 μL HEPES buffer, Condition B: 150 μL HEPES buffer + 50 μL NPN 40 μM, Condition C: 100 μL HEPES buffer + 100 μL bacteria, Condition D (in triplicate): 50 μL HEPES buffer + 100 μL bacteria + 50 μL NPN 40 μM and Condition E: 200 μL bacteria. The NPN was added last to the indicated wells, just before measuring fluorescence (360 nm excitation, 405 nm emission) and the OD_405_ with a microplate reader (Spectramax Paradigm from Molecular Devices). The signal was measured every 1.5 min for 30 min and the mean of all the measurements was calculated. For each sample, the fluorescence (arbitrary units; A.U.) was divided by the OD_405_ to obtain the corrected fluorescence value.

#### Resistance of *P. aeruginosa* to H_2_O_2_

To determine the sensitivity of *P. aeruginosa* to H_2_O_2_, 200 CFU of *P. aeruginosa* were incubated with various concentrations of H_2_O_2_ (5–100 mM) in 50 μL of PB buffer for 30 min at room temperature under agitation. Then 600 μL of LB medium was added to the tube and 100 μL of the resulting suspension was plated on LB-agar and incubated at 37°C overnight before counting colonies. The same experiment was also carried out using a fixed concentration of H_2_O_2_ (50mM).

#### Characterization of *P. aeruginosa* LPS

Two methods were used to characterize *P. aeruginosa* LPS: immunostaining of surface LPS, and analysis of purified LPS.

To stain the bacterial surface LPS, we used the AE404 antibody (ABCD nomenclature, http://web.expasy.org/abcd/) as previously described.^15^ The AE404 antibody was produced as mini-antibodies with the antigen-binding portion fused to a mouse IgG2A. Bacteria were pelleted (6000 g, 3 min), washed once and resuspended in PB-Sorbitol, and diluted 1/100. A shaken suspension of diluted bacteria (200 μL) was incubated for 10 min with the AE404 antibody (2 μg/mL) at room temperature, then centrifuged, washed once and incubated for 20 min in PB-Sorbitol containing Alexa 488-coupled goat anti-mouse IgG (Life Technologies A11029, diluted 1/200). Bacteria were washed once and resuspended in 400 μL of PB-Sorbitol before analysis by flow cytometry (BD LSR Fortessa Cell Analyzer, 647800E6).

We purified and visualized *P. aeruginosa* LPS as previously described^20^ using an LPS purification kit (17141, iNtRON Biotechnology) and following manufacturer’s instructions. Briefly, bacteria (350 μL) were pelleted (6000 g, 3 min), then lysed in an organic solution containing phenol (800 μL). Chloroform (200 μL) was added to remove lipids, proteins amd nucleic acids from the extract, and the aqueous phase collected (400 μL). LPS were then precipitated from the aqueous phase at high salt concentration, pelleted (15,’000 g, 15 min, 4°C), washed with 70% EtOH, and resuspended in 10mM Tris-HCl buffer (pH 8.0). To visualize them, purified LPS were separated in a 4-15% Mini-PROTEAN1TGX™ Precast protein gel (BioRad) then silver-stained: the gel was rinsed with water (3 × 5 min), fixed in 50 mL fixation solution (30 min; 40% ethanol, 10% acetic acid, 50% water), incubated overnight in 50 mL of a freshly-made pre-staining solution (15 mL ethanol, 25 mL water, 130 μL glutaraldehyde (50% in water), 10mL of sensitizing solution (2.5 M sodium acetate, 40 mM sodium thiosulfate in water). The gel was then washed with water (3 × 5 min) and incubated for 40 min in 50 mL of freshly-made silver nitrate solution (50 mg silver nitrate, 50mL water, 46 μL formaldehyde (36% in water). Finally, the gel was rinsed once in water, once in 50 mL development solution (100 mL 0.25 M NaCO3 in water, 10 μL formaldehyde) and incubated in 50 mL development solution until the appearance of the LPS profiles (approximately 15 min). The development was stopped in 50 mL of 40 mM EDTA.

### Quantification and statistical analysis

As indicated in each figure legend, to perform statistical analysis, we used the results of at least 5 independent experiments (N), and made use of non-parametric tests (two data sets: Mann-Whitney, unpaired (respectively, paired) multiple data sets: Kruskal–Wallis (respectively, Friedman) test followed by Dunn's multiple comparison. Stars indicate statistically significant differences between samples (*p* <0.05).

## Supplemental information

Document S1. Figures S1–S4

Table S1. Detailed proteomic analysis of *D. discoideum* secreted proteins

## References

[1] Adiba S., Nizak C., van Baalen M., Denamur E., Depaulis F. (2010). From grazing resistance to pathogenesis: the coincidental evolution of virulence factors. PLoS One.

[2] Casadevall A., Pirofski L.A. (2007). Accidental virulence, cryptic pathogenesis, martians, lost hosts, and the pathogenicity of environmental microbes. Eukaryot. Cell.

[3] Price C.T.D., Hanford H.E., Al-Quadan T., Santic M., Shin C.J., Da'as M.S.J., Abu Kwaik Y. (2024). Amoebae as training grounds for microbial pathogens. mBio.

[4] Hassett D.J., Ma J.F., Elkins J.G., McDermott T.R., Ochsner U.A., West S.E., Huang C.T., Fredericks J., Burnett S., Stewart P.S. (1999). Quorum sensing in *Pseudomonas aeruginosa* controls expression of catalase and superoxide dismutase genes and mediates biofilm susceptibility to hydrogen peroxide. Mol. Microbiol..

[5] O'Toole G.A. (2018). Cystic Fibrosis Airway Microbiome: Overturning the Old, Opening the Way for the New. J. Bacteriol..

[6] Jurado-Martín I., Sainz-Mejías M., McClean S. (2021). *Pseudomonas aeruginosa*: An Audacious Pathogen with an Adaptable Arsenal of Virulence Factors. Int. J. Mol. Sci..

[7] Swain J., Askenasy I., Rudland Nazeer R., Ho P.M., Labrini E., Mancini L., Xu Q., Hollendung F., Sheldon I., Dickson C. (2025). Pathogenicity and virulence of *Pseudomonas aeruginosa*: Recent advances and under-investigated topics. Virulence.

[8] Zhang X., Zhang D., Zhou D., Zheng S., Li S., Hou Q., Li G., Han H. (2025). A comprehensive review of the pathogenic mechanisms of *Pseudomonas aeruginosa*: synergistic effects of virulence factors, quorum sensing, and biofilm formation. Front. Microbiol..

[9] Dunn J.D., Bosmani C., Barisch C., Raykov L., Lefrançois L.H., Cardenal-Muñoz E., López-Jiménez A.T., Soldati T. (2017). Eat Prey, Live: *Dictyostelium discoideum* As a Model for Cell-Autonomous Defenses. Front. Immunol..

[10] Cosson P., Soldati T. (2008). Eat, kill or die: when amoeba meets bacteria. Curr. Opin. Microbiol..

[11] Jauslin T., Lamrabet O., Crespo-Yañez X., Marchetti A., Ayadi I., Ifrid E., Guilhen C., Leippe M., Cosson P. (2021). How Phagocytic Cells Kill Different Bacteria: a Quantitative Analysis Using *Dictyostelium discoideum*. mBio.

[12] Cosson P., Zulianello L., Join-Lambert O., Faurisson F., Gebbie L., Benghezal M., Van Delden C., Curty L.K., Köhler T. (2002). *Pseudomonas aeruginosa* virulence analyzed in a *Dictyostelium discoideum* host system. J. Bacteriol..

[13] Pukatzki S., Kessin R.H., Mekalanos J.J. (2002). The human pathogen *Pseudomonas aeruginosa* utilizes conserved virulence pathways to infect the social amoeba *Dictyostelium discoideum*. Proc. Natl. Acad. Sci. USA.

[14] Ayadi I., Lamrabet O., Munoz-Ruiz R., Jauslin T., Guilhen C., Cosson P. (2024). Extracellular and intracellular destruction of *Pseudomonas aeruginosa* by *Dictyostelium discoideum* phagocytes mobilize different antibacterial mechanisms. Mol. Microbiol..

[15] Ayadi I. (2021). The AE404 antibody recognizes a *Pseudomonas aeruginosa* PAO1 surface antigen by flow cytometry. Antib. Rep..

[16] Lam J.S., Taylor V.L., Islam S.T., Hao Y., Kocíncová D. (2011). Genetic and Functional Diversity of *Pseudomonas aeruginosa* Lipopolysaccharide. Front. Microbiol..

[17] Cornillon S., Pech E., Benghezal M., Ravanel K., Gaynor E., Letourneur F., Brückert F., Cosson P. (2000). Phg1p is a nine-transmembrane protein superfamily member involved in dictyostelium adhesion and phagocytosis. J. Biol. Chem..

[18] He J., Baldini R.L., Déziel E., Saucier M., Zhang Q., Liberati N.T., Lee D., Urbach J., Goodman H.M., Rahme L.G. (2004). The broad host range pathogen *Pseudomonas aeruginosa* strain PA14 carries two pathogenicity islands harboring plant and animal virulence genes. Proc. Natl. Acad. Sci. USA.

[19] Gilbert K.B., Kim T.H., Gupta R., Greenberg E.P., Schuster M. (2009). Global position analysis of the *Pseudomonas aeruginosa* quorum-sensing transcription factor LasR. Mol. Microbiol..

[20] Ifrid E., Ouertatani-Sakouhi H., Jauslin T., Kicka S., Chiriano G., Harrison C.F., Hilbi H., Scapozza L., Soldati T., Cosson P. (2022). 5-ethyl-2'-deoxyuridine fragilizes Klebsiella pneumoniae outer wall and facilitates intracellular killing by phagocytic cells. PLoS One.

[21] Helander I.M., Mattila-Sandholm T. (2000). Fluorometric assessment of gram-negative bacterial permeabilization. J. Appl. Microbiol..

[22] Munoz-Ruiz R., Lamrabet O., Jauslin T., Guilhen C., Bourbon A., Cosson P. (2024). Antibacterial effectors in *Dictyostelium discoideum*: specific activity against different bacterial species. mSphere.

[23] Guilhen C., Lima W.C., Ifrid E., Crespo-Yañez X., Lamrabet O., Cosson P. (2020). A New Family of Bacteriolytic Proteins in *Dictyostelium discoideum*. Front. Cell. Infect. Microbiol..

[24] Vogel G., Thilo L., Schwarz H., Steinhart R. (1980). Mechanism of phagocytosis in *Dictyostelium discoideum*: phagocytosis is mediated by different recognition sites as disclosed by mutants with altered phagocytotic properties. J. Cell Biol..

[25] Pan M., Neilson M.P., Grunfeld A.M., Cruz P., Wen X., Insall R.H., Jin T. (2018). A G-protein-coupled chemoattractant receptor recognizes lipopolysaccharide for bacterial phagocytosis. PLoS Biol..

[26] Lima W.C., Balestrino D., Forestier C., Cosson P. (2014). Two distinct sensing pathways allow recognition of Klebsiella pneumoniae by Dictyostelium amoebae. Cell. Microbiol..

[27] Cornillon S., Gebbie L., Benghezal M., Nair P., Keller S., Wehrle-Haller B., Charette S.J., Brückert F., Letourneur F., Cosson P. (2006). An adhesion molecule in free-living Dictyostelium amoebae with integrin beta features. EMBO Rep..

[28] Sattler N., Bosmani C., Barisch C., Guého A., Gopaldass N., Dias M., Leuba F., Bruckert F., Cosson P., Soldati T. (2018). Functions of the Dictyostelium LIMP-2 and CD36 homologues in bacteria uptake, phagolysosome biogenesis and host cell defence. J. Cell Sci..

[29] Huszcynski S.M., Lam J.S., Khursigara C.M. (2019). The role of Pseudomonas aeruginosa lipopolysaccharide in bacterial pathogenesis and physiology. Pathogens.

[30] Gerber-Tichet E., Blanchet F.P., Majzoub K., Kremer E.J. (2025). Toll-like receptor 4 - a multifunctional virus recognition receptor. Trends Microbiol..

[31] Engels W., Endert J., Kamps M.A., van Boven C.P. (1985). Role of lipopolysaccharide in opsonization and phagocytosis of *Pseudomonas aeruginosa*. Infect. Immun..

[32] Demirdjian S., Schutz K., Wargo M.J., Lam J.S., Berwin B. (2017). The effect of loss of O-antigen ligase on phagocytic susceptibility of motile and non-motile *Pseudomonas aeruginosa*. Mol. Immunol..

[33] Maldonado R.F., Sá-Correia I., Valvano M.A. (2016). Lipopolysaccharide modification in Gram-negative bacteria during chronic infection. FEMS Microbiol. Rev..

[34] Lelong E., Marchetti A., Simon M., Burns J.L., van Delden C., Köhler T., Cosson P. (2011). Evolution of *Pseudomonas aeruginosa* virulence in infected patients revealed in a *Dictyostelium discoideum* host model. Clin. Microbiol. Infect..

[35] Köhler T., Buckling A., van Delden C. (2009). Cooperation and virulence of clinical *Pseudomonas aeruginosa* populations. Proc. Natl. Acad. Sci. USA.

[36] Froquet R., Lelong E., Marchetti A., Cosson P. (2009). *Dictyostelium discoideum*: a model host to measure bacterial virulence. Nat. Protoc..

[37] Köhler T., Curty L.K., Barja F., van Delden C., Pechère J.C. (2000). Swarming of *Pseudomonas aeruginosa* is dependent on cell-to-cell signaling and requires flagella and pili. J. Bacteriol..

[38] Bélanger M., Burrows L.L., Lam J.S. (1999). Functional analysis of genes responsible for the synthesis of the B-band O antigen of *Pseudomonas aeruginosa* serotype O6 lipopolysaccharide. Microbiology.

[39] Thibault J., Faudry E., Ebel C., Attree I., Elsen S. (2009). Anti-activator ExsD forms a 1:1 complex with ExsA to inhibit transcription of type III secretion operons. J. Biol. Chem..

[40] Crespo-Yanez X., Oddy J., Lamrabet O., Jauslin T., Marchetti A., Cosson P. (2023). Sequential action of antibacterial effectors in *Dictyostelium discoideum* phagosomes. Mol. Microbiol..

[41] Marchetti A., Lima W.C., Hammel P., Cosson P. (2023). A quantitative comparison of antibodies against epitope tags for immunofluorescence detection. FEBS Open Bio.

