# Supplementary material for: LPS and quorum sensing protect Pseudomonas aeruginosa bacteria from phagocytosis and destruction by Dictyostelium discoideum amoebae
Source: iScience. 2026 Oct 19;29(11):117912. doi: 10.1016/j.isci.2026.117912 (PMC13695109; doi:10.1016/j.isci.2026.117912)
Supplement: Supplementary file 1 [file mmc1.pdf]

iScience, Volume *29*

Supplemental information

LPS and quorum sensing protect *Pseudomonas aeruginosa* bacteria from phagocytosis and destruction by *Dictyostelium discoideum* amoebae

Imen Ayadi, Erwann Pain, Joseph Oddy, Otmane Lamrabet, Hitakshi Vijay, Thilo Koehler, Tania Distler, Alexandre Persat, Raphael Munoz-Ruiz, and Pierre Cosson

SUPPLEMENTAL FIGURES

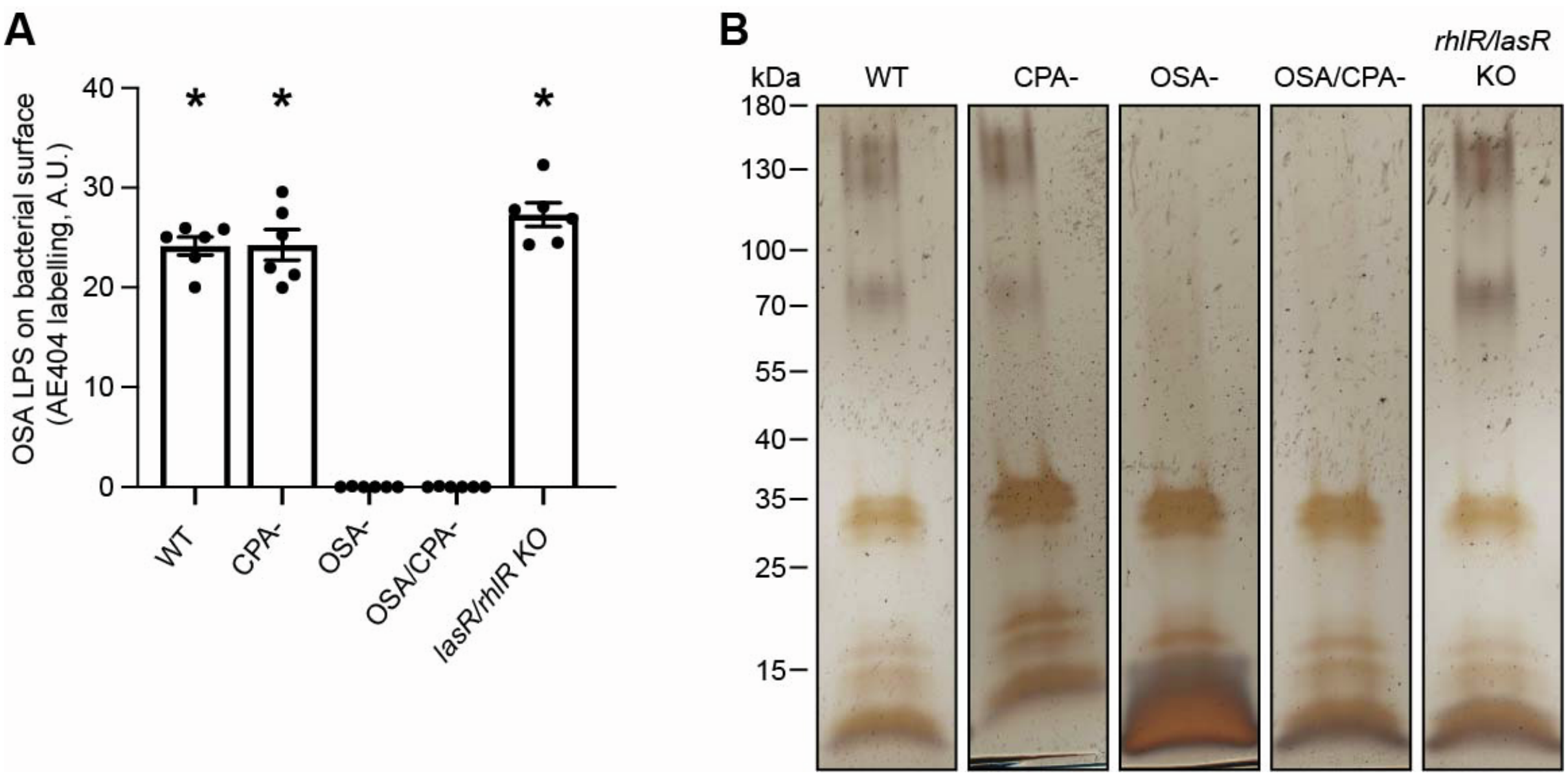

Figure S1 – Detection of OSA-type LPS in PAO1 strains. A. Related to Figure 1C. *P. aeruginosa* PAO1 (WT) and mutants (defective for CPA LPS: CPA-; defective for OSA-type LPS: OSA-) were tested for expression of OSA-type LPS by staining their surface LPS with the AE404 antibody. WT, CPA- and lasR/rhlR exhibited a positive signal; *p < 0.05; Kruskal–Wallis test followed by Dunn's multiple comparison, N=6 independent experiments. **B.** The LPS of each strain were purified and migrated on an SDS-PAGE gel. Both methods reveal the absence of OSA-type LPS with extended O-antigen side chains in OSA- strains. On the contrary, the genetic inactivation of the Las and Rhl QS did not visibly alter the OSA-type LPS.

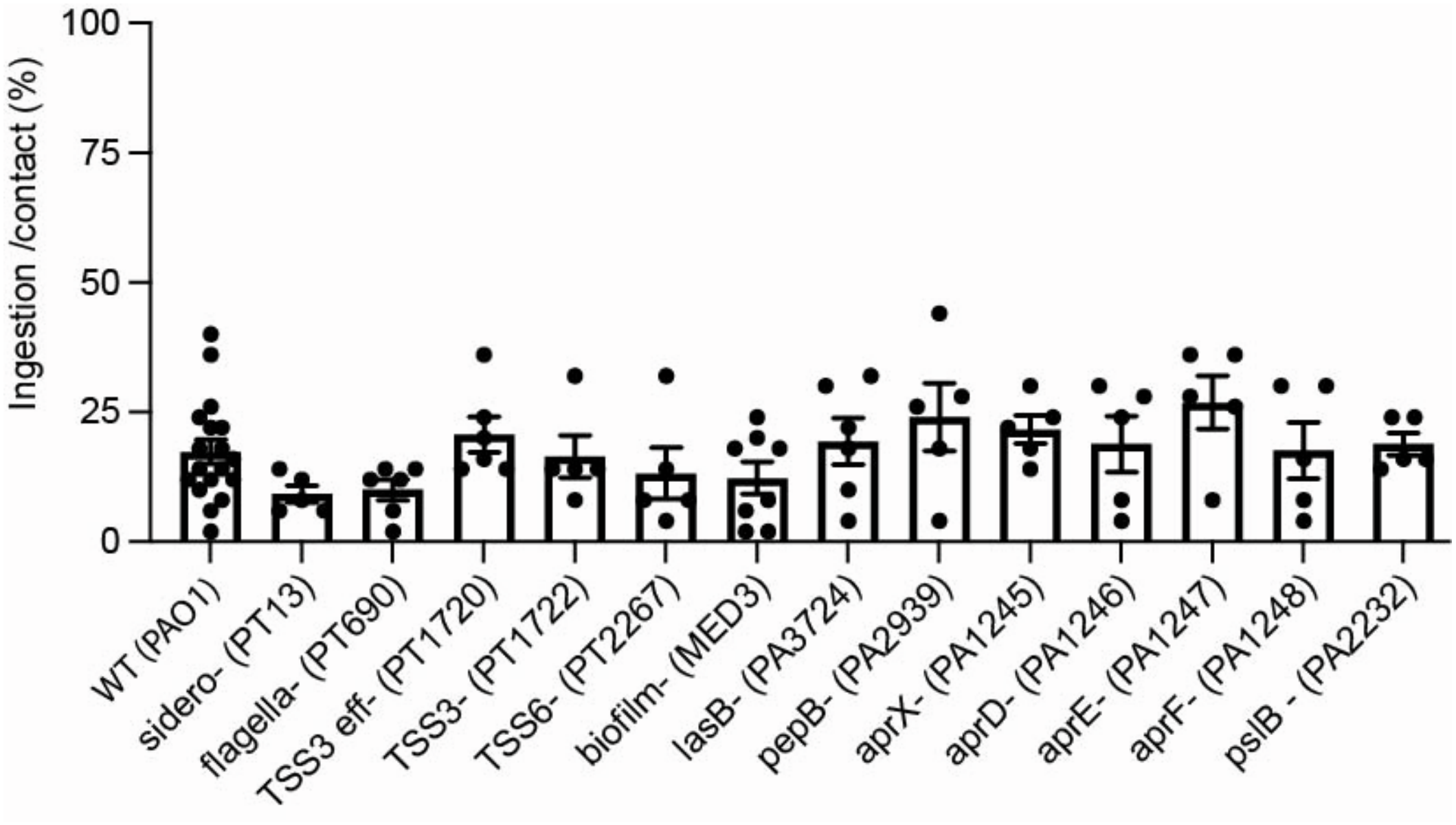

Figure S2 – Resistance of *P. aeruginosa* mutants to phagocytosis by *D. discoideum*. Related to Figure 1C. *P. aeruginosa* mutants lacking different putative virulence genes were tested for their resistance to phagocytosis by *D. discoideum* as described in Figure 1. The strains used in this figure are described in Table 1. None of the mutant strains analysed was phagocytosed significantly more than WT *P. aeruginosa*. N=5 independent experiments, Kruskal–Wallis test ($p < 0.05$) followed by Dunn's multiple comparison test.

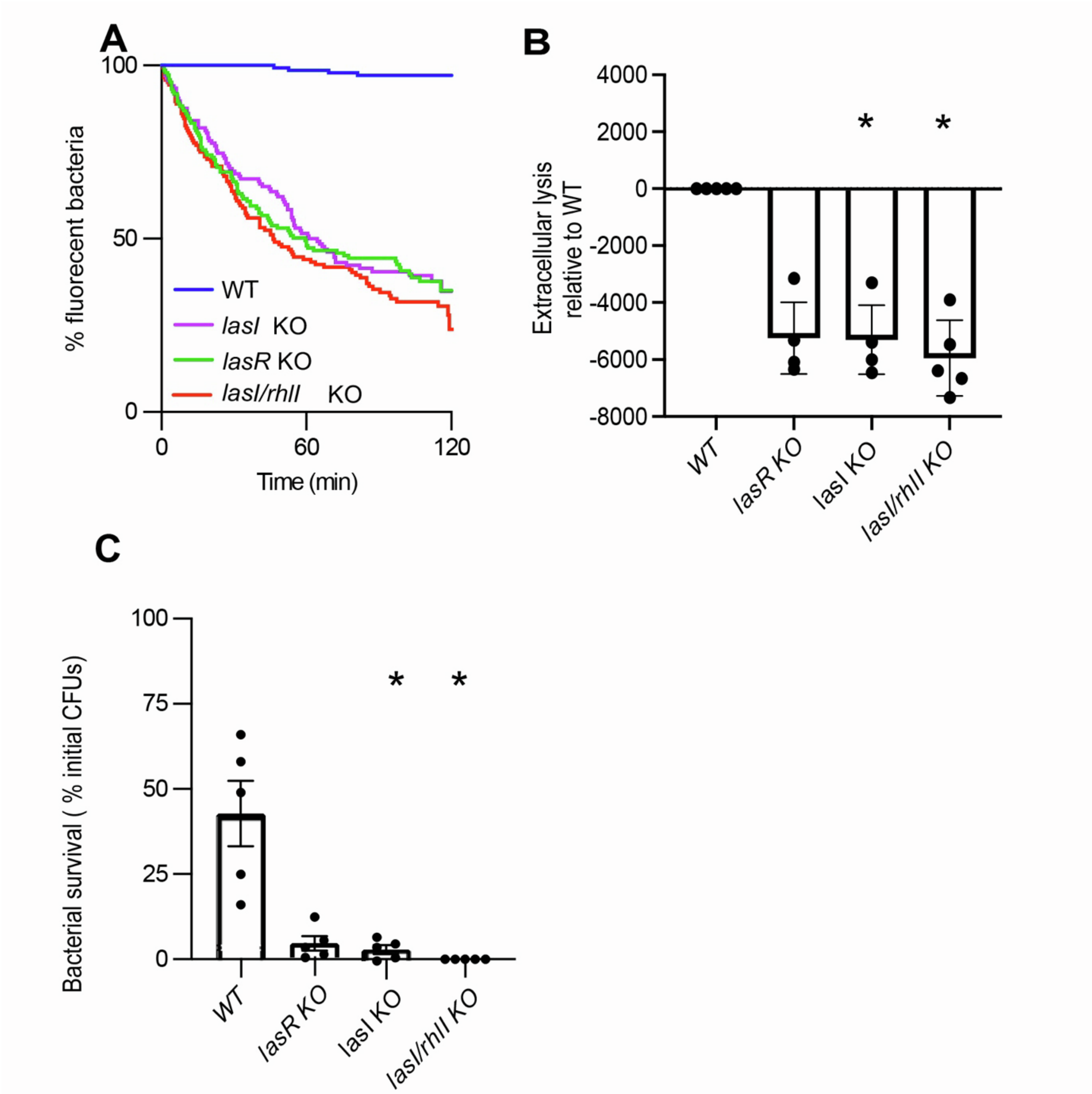

Figure S3 – Genetic inactivation of LasI renders *P. aeruginosa* sensitive to H_2O_2 and to extracellular lysis by *D. discoideum*. Related to Figure 4. A. Survival of extracellular *P. aeruginosa* following contact with *D. discoideum* was determined as described in Figure 4. For each bacteria analysed, time 0 indicates the time when it was touched by the amoeba and GFP-extinction is concomitant with bacterial lysis. The cumulative survival curve of bacteria is shown. Mutants lacking LasI QS were significantly more lysed than WT bacteria. (n=150 bacteria; N = 5 independent

experiments). **B.** The area under the survival curve was determined in individual experiments and normalized to WT. Mutant bacteria lacking LasI QS were lysed extracellularly faster than WT bacteria. mean ± SEM. * $p < 0.05$; Kruskal–Wallis test followed by Dunn's multiple comparison test, N=5 independent experiments. **C.** *P. aeruginosa* were incubated with 50mM H_2O_2 for 30 min then plated on LB-agar to determine their viability, as described in Figure 5. Mutants of *P. aeruginosa* lacking LasI QS were more efficiently killed by H_2O_2 than WT bacteria. Friedman test followed by Dunn's multiple comparison, *: $p<0.05$, Kruskal–Wallis test followed by Dunn's multiple comparison test, N=5 independent experiments. CFU: colony forming unit.

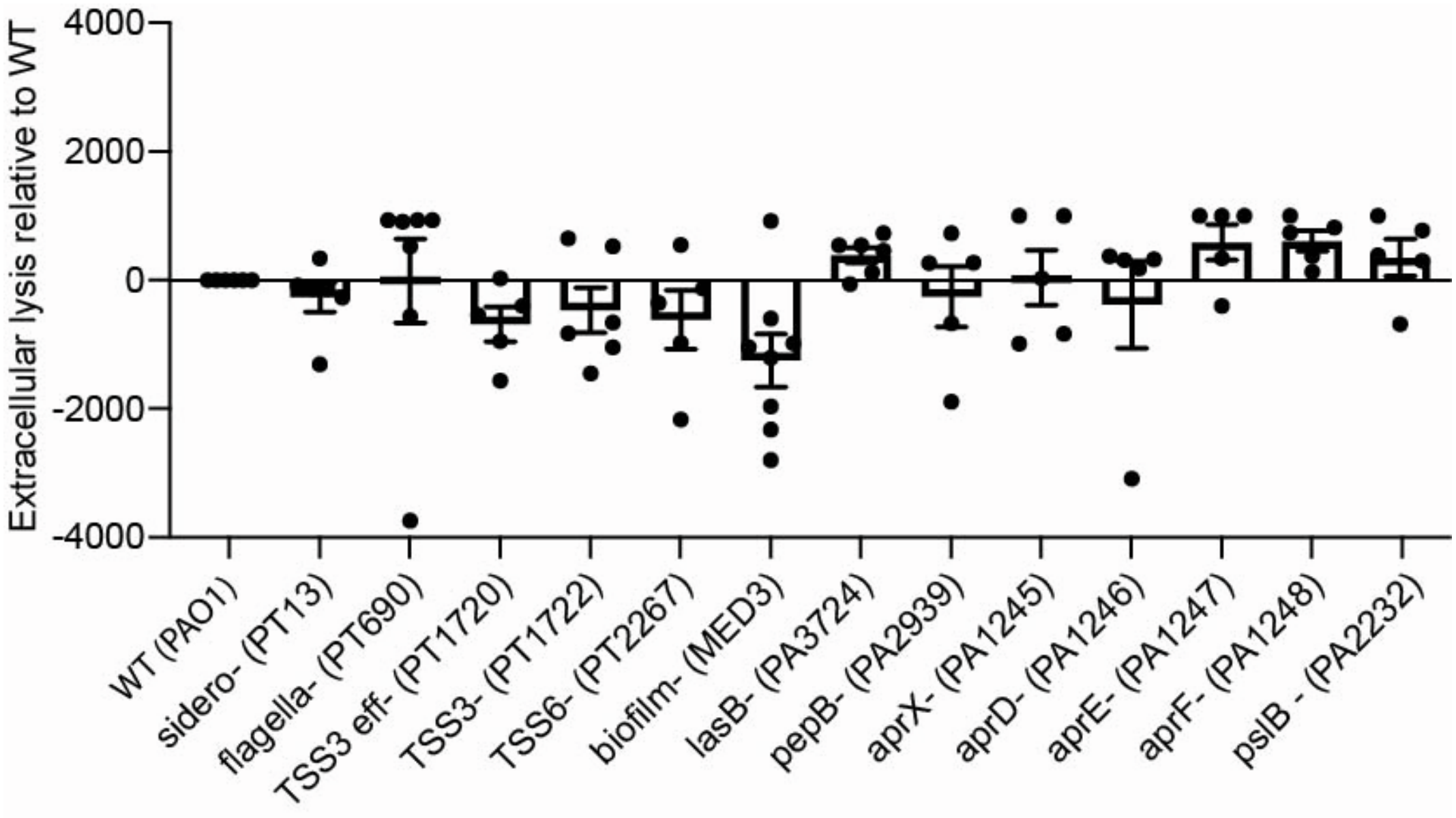

Figure S4 – Resistance of *P. aeruginosa* mutants to extracellular lysis by *D. discoideum*. Related to Figure 4. *P. aeruginosa* mutants lacking different putative virulence genes were tested for their resistance to extracellular lysis by *D. discoideum* as described in Figure 4. The strains used in this figure are described in Table 1. None of the mutant strains analysed was lysed significantly faster than WT *P. aeruginosa*. N=5 independent experiments, Kruskal–Wallis test ($p < 0.05$) followed by Dunn's multiple comparison test.
